# Transcranial Magnetic Stimulation as a Potential Biomarker in Multiple Sclerosis: A Systematic Review with Recommendations for Future Research

**DOI:** 10.1155/2019/6430596

**Published:** 2019-09-16

**Authors:** Nicholas J. Snow, Katie P. Wadden, Arthur R. Chaves, Michelle Ploughman

**Affiliations:** Faculty of Medicine, Memorial University of Newfoundland, St. John's, NL, Canada

## Abstract

Multiple sclerosis (MS) is a demyelinating disorder of the central nervous system. Disease progression is variable and unpredictable, warranting the development of biomarkers of disease status. Transcranial magnetic stimulation (TMS) is a noninvasive method used to study the human motor system, which has shown potential in MS research. However, few reviews have summarized the use of TMS combined with clinical measures of MS and no work has comprehensively assessed study quality. This review explored the viability of TMS as a biomarker in studies of MS examining disease severity, cognitive impairment, motor impairment, or fatigue. Methodological quality and risk of bias were evaluated in studies meeting selection criteria. After screening 1603 records, 30 were included for review. All studies showed high risk of bias, attributed largely to issues surrounding sample size justification, experimenter blinding, and failure to account for key potential confounding variables. Central motor conduction time and motor-evoked potentials were the most commonly used TMS techniques and showed relationships with disease severity, motor impairment, and fatigue. Short-latency afferent inhibition was the only outcome related to cognitive impairment. Although there is insufficient evidence for TMS in clinical assessments of MS, this review serves as a template to inform future research.

## 1. Introduction

Multiple sclerosis (MS) is a neuroimmune-regulated demyelinating disease affecting the central nervous system (CNS) [1, 2]. Although the exact etiology of MS is not fully understood, disease pathophysiology is characterized by a process of blood-brain barrier damage, inflammation involving chiefly macrophages and microglia, demyelination of gray and white matter, loss of oligodendrocytes, reactive gliosis in parenchymal tissue, axonal degeneration and transection, and cortical atrophy [2]. This process is thought to be incited by environmental triggers in a genetically susceptible individual, albeit both the suspected environmental factors and candidate genes are numerous [1, 2].

Throughout the course of MS, damage to gray and white matter [1–4] contributes to deficits such as visual impairment [5], cognitive impairment [6], motor impairment [7], and fatigue [8–10]. These signs and symptoms represent some of the most common nonpsychiatric features in MS [11–13] and significantly impact individuals' quality of life and ability to participate in society [14]. Clinical presentation in MS is highly variable, and disease progression is unpredictable [11, 13, 15–17]. However, changes in myelination can be observed early in the disease, prior to the emergence of clinical findings [2, 18, 19]. Indeed, overt and subtle gray and white matter damage occurs from earliest disease stages [20–22] and without early intervention and many individuals experience permanent disability as the disease progresses [3, 11, 14, 16, 23]. Taken together, the above evidence emphasizes the urgent need to establish viable biological markers (“biomarkers”) of disease status in MS [2, 24].

Applications of biomarkers include use as a diagnostic tool, classifying the extent of a disease, indicating disease prognosis, and predicting and monitoring clinical response to an intervention [25]. At present, there are few biomarkers for the clinical evaluation of MS [26, 27]. Differentiation between relapsing-remitting (RRMS) and progressive subtypes of MS—disease stages with markedly different pathophysiology [2]—is based almost solely on clinical features, and few reliable biomarkers of disease progression have been established to help guide treatments [26, 27]. Nevertheless, several prospective modalities are under continuing evaluation and validation, including magnetic resonance imaging (MRI) [28, 29], optical coherence tomography (OCT) [30], corticospinal fluid (CSF) parameters [31], and neurofilament light chain (NfL) analyses [32]. Alternatively, some work has argued that TMS may be ideally suited as a surrogate marker for MS [33–35]. TMS has the potential to be less expensive, time consuming, and invasive than other methodologies used in the clinical approach to MS, lending support to its clinical use [36]. Furthermore, TMS has the unique ability to map and interrogate, in real time, characteristics of the CNS such as corticomotor latency, intracortical excitability, and transcallosal inhibition, which can be examined in relation to observable behaviour and clinical signs [24, 33–35, 37]. However, some TMS measures can be unreliable both between individuals and across time [38, 39]; their utility is highly dependent on factors related to the research participant, disease etiology, and laboratory environment [34, 40]; and stringent controls and rigorous reporting are required to glean valid physiological and clinical information from TMS findings [40]. As well, studies vary in sample size, participant characteristics, and reporting of results [34]. Therefore, TMS literature examining persons with MS could be susceptible to risk of bias. Finally, few works have combined TMS and clinical assessments of MS [8, 41–43]. Overall, it is challenging to determine the clinical utility of TMS in its application to MS [33, 34, 44].

To advance the role of TMS as a biomarker in clinical assessments of MS, the viability of this technique for this purpose must be better established through the interrogation of study quality. To this end, we conducted a systematic review of the literature evaluating cross-sectional comparisons of TMS and clinical outcomes in persons with MS and healthy controls (HC). While similar reviews have been conducted for other neurological illnesses [45, 46], this is the first such review performed in MS. Our objectives were (i) to systematically assess the methodological quality and risk of bias in studies of TMS and clinical outcomes of disease severity, cognitive impairment, motor impairment, or fatigue in MS and (ii) to synthesize the findings of these studies, including relationships between TMS and clinical outcomes.

## 2. Materials and Methods

The review protocol was registered in the PROSPERO International Prospective Register of Systematic Reviews (ID: CRD42017082333). We sought to examine research studies involving cross-sectional comparisons of adult human persons with MS and HC participants. We aimed to include studies involving cross-sectional comparisons of participant groups, using single- or paired-pulse TMS in combination with clinical measures of disease severity and cognitive impairment, motor impairment, or fatigue impact and severity. These outcomes were used to explore relationships between TMS findings and clinical features of MS. Types of acceptable studies included cross-sectional observational studies, as well as baseline data from interventional or longitudinal studies. We examined study quality and risk of bias based on aspects of study design, methodology, and reporting, including key confounding variables such as participant factors (e.g., age, sex, and disease status), drug and medication factors, and technical considerations (e.g., TMS parameters).

### 2.1. Search Strategy

We electronically searched the Web of Science, MEDLINE, and Embase databases for studies published between January 1, 1985 (the first year of TMS publication [47]) and September 8, 2017. The search was repeated on November 29, 2018. The following search terms were used: “multiple sclerosis” AND (“transcranial magnetic stimulation” OR “tms” OR “magnetic stimulation”). We selected studies that met above conditions, and we manually searched for studies examining outcomes of interest, to increase the number of search hits. Search results were imported into Microsoft Excel (V2016, Microsoft Corporation, Redmond, WA, USA). All article screenings were conducted in Excel. Prior to study selection, all duplicate records were removed. The review was conducted by two independent raters (KPW and NJS). All discrepancies and uncertainties were resolved by consensus.

### 2.2. Study Selection

The following selection criteria were used to screen articles. It was required that articles (1) be reported in full-text peer-reviewed manuscripts, published in English; (2) compare adult (age ≥ 18 yr) human participants with a primary diagnosis of MS and HC participants; (3) include a diagnosis of MS that is definite and explicitly based on Poser [48], McDonald [23], or revised McDonald [49, 50] criteria; (4) utilize TMS for measurement purposes (i.e., no plasticity-inducing protocols such as repetitive TMS or paired associative stimulation), in combination with validated clinical measures of disease severity and cognitive impairment, motor impairment, or fatigue; (5) report one or more of the above clinical scales as outcome measures; (6) use the Expanded Disability Status Scale (EDSS) [51] to index MS participants' disease severity; (7) use surface EMG measurements from upper limb muscles, in conjunction with TMS delivered over a scalp site; and (8) be observational and cross-sectional or include baseline statistical comparisons of MS and HC groups in the case of interventional or longitudinal studies.

#### 2.2.1. Title and Abstract Review

Titles and abstracts of nonduplicate search results were weighed against inclusion and exclusion criteria by two independent raters (KPW and NJS).

#### 2.2.2. Full-Text Review

All articles passing the title and abstract review were read in full and further screened against inclusion and exclusion criteria. In any case where a full-text article could not be obtained, the corresponding author of that study was contacted to request the manuscript. Reference lists of relevant review articles were scanned for additional records, which were then screened as above. For items that passed this review stage, their reference lists were searched to identify additional relevant studies, which were then likewise screened. Any discrepancies were addressed by consensus. All items finally included after this review stage underwent risk of bias assessment and data extraction. Interrater agreement was determined using Cohen's kappa (*κ*) [52]. Values were interpreted as no agreement (<0.20), minimal agreement (0.21-0.39), weak agreement (0.40-0.59), moderate agreement (0.60-0.79), strong agreement (0.80-0.90), and almost perfect agreement (>0.90) [52].

### 2.3. Risk of Bias Assessment

The risk of bias assessment was performed by two raters independently (KPW and NJS), and discrepancies were resolved by consensus. A modified version of the National Institutes of Health (NIH) “Quality Assessment Tool for Observational Cohort and Cross-Sectional Studies” was used to examine study quality and risk of bias [53]. Criteria for risk of bias are listed in Table 1. For key confounding variables (criterion 14), we gathered a list of factors deemed important for the investigations of interest, based on the extant literature [40, 54–61]. This list of key confounding variables is shown in Table 2. To guide decisions on overall study quality from the NIH tool [53], the Cochrane Risk of Bias Tool was used [62]. An article was deemed to have a high risk of bias (i.e., low quality) if one or more criteria from the NIH tool was unmet and marked “N,” unclear risk (i.e., moderate quality/risk) if one or more criteria were ambiguous and marked “U” and no criterion was marked “N,” and high quality (i.e., low risk) if all 14 criteria were clearly met and marked “Y”.

### 2.4. Data Extraction

Data extraction was performed by two raters independently (KPW and NJS). All disagreements were resolved by consensus. Data retrieved included participant characteristics (i.e., sample or subsample (e.g., MS subtype) size, sex, age, disease duration, and EDSS score), routine treatment for MS participants (i.e., type of drug and number of participants), cortical target for TMS, TMS coil type (i.e., geometry and diameter), TMS protocols utilized, clinical measures examined, statistically significant TMS findings, statistically significant clinical findings, and statistically significant correlations between clinical and TMS data. Findings were reported as changes in MS versus HC samples or as nonsignificant, with *p* values provided where possible. The strength and direction of significant correlations were reported when possible.

## 3. Results

### 3.1. Study Selection

**Figure 1:**
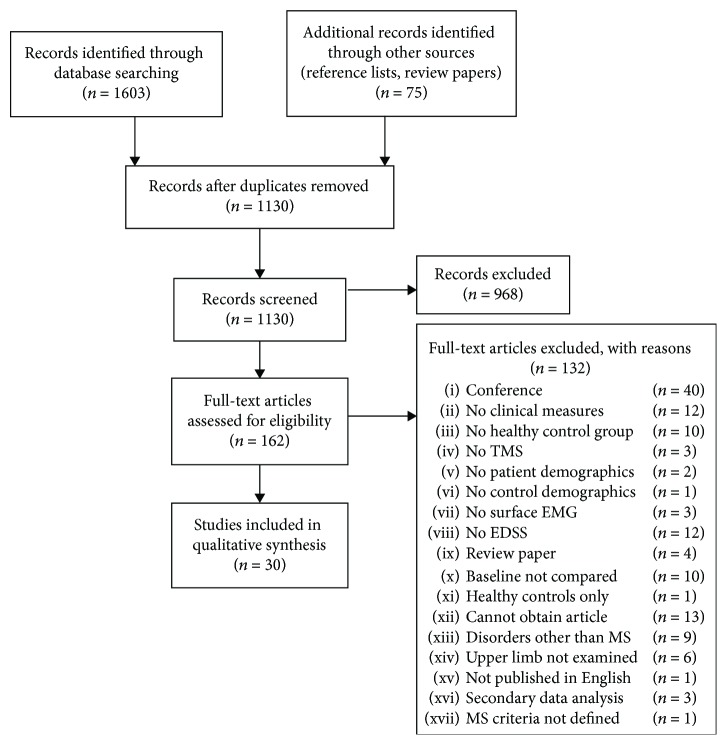
Flow chart detailing study screening.

The progression of article inclusion and exclusion is shown in Figure 1. Our electronic database searches yielded 1603 records, plus an additional 75 items from reference lists of review articles (*n* = 42), reference lists of included full texts (*n* = 24), and manual webpage searches (*n* = 9). After removing duplicates, there were 1130 records remaining for title and abstract review. Following title and abstract review, 162 items were included for full-text review. Of those 162 records, a total of 30 articles were finally retained for risk of bias assessment and data extraction. Interrater agreement (Cohen's *κ*) ranged from 0.46 to 0.77 (weak to moderate agreement), prior to reaching consensus. Consensus was reached at all review stages.

### 3.2. Risk of Bias Assessment

**Figure 2:**
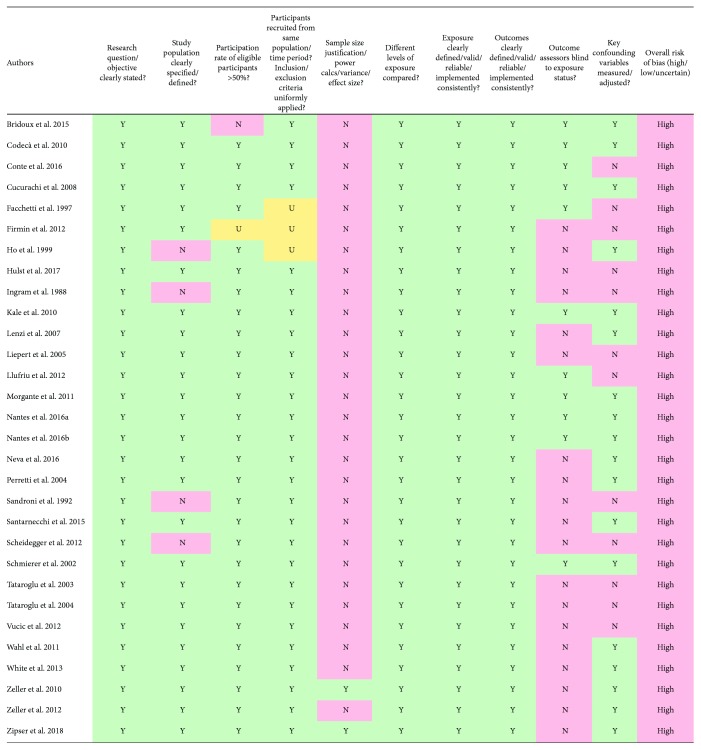
Results of study quality and risk of bias assessment. Risk of bias assessment was conducted using a modified version of the National Institutes of Health (NIH) “Quality Assessment Tool for Observational Cohort and Cross-Sectional Studies” [53]. To guide decisions on overall study quality from the NIH tool [53], the Cochrane Risk of Bias Tool was used [62]. An article was deemed to have a high risk of bias (i.e., low quality) if one or more criteria from the NIH tool was unmet and marked “N,” unclear risk (i.e., moderate quality/risk) if one or more criteria were ambiguous and marked “U” and no criterion was marked “N”, and high quality (i.e., low risk) if all 14 criteria were clearly met and marked “Y.” Key confounding variables can be found in [Supplementary-material supplementary-material-1].

Based on the quality assessment of methodologies employed in the present review, all 30 studies were deemed to be at a high risk of bias (Figure 2).

#### 3.2.1. Risk of Bias Criteria

When examining sources of possible bias, the greatest contributor was criterion 5 (i.e., sample size justification, power description, or variance and effect size estimate); only two records provided justification for the sample size employed [63, 64]. The next greatest cause of potential bias was criterion 12 (i.e., blinding of outcome assessors). Eleven articles employed rater blinding during at least one study component [8, 42, 43, 54, 65–71]. The third largest area of anticipated bias was criterion 14 (i.e., key potential confounding variables). Eighteen studies adequately accounted for potential confounds [42, 43, 63–67, 69–79].

#### 3.2.2. Confounding Factors

Eighteen studies sufficiently accounted for key confounding variables, having addressed at least 19 sources of potential confounding ([Supplementary-material supplementary-material-1]) [42, 43, 63–67, 69–79]. In terms of key confounding variables not well controlled for, no studies accounted for participants' history of specific repetitive motor activity (criterion viii). Only one study controlled for the participants' attention level during TMS (criterion xxiii) [64]. Similarly, one study accounted for ingestion of nonprescription drugs shown to influence responses to TMS (criterion xxxvii) [64]. Regarding well-controlled potential confounding variables, all 30 studies reported a metric of MS disease severity (criterion xxxii), as well as a method for determining MEP size (criterion xxix). As well, where applicable all studies reported parameters for paired-pulse TMS (i.e., criterion xxvi, test pulse intensity; criterion xxvii, conditioning pulse intensity; and criterion xxviii, interstimulus interval).

### 3.3. Data Extraction

Detailed results can be found in [Supplementary-material supplementary-material-1]. Of the 30 articles included in the final data synthesis, all considered a TMS-based measure targeting the cortical representation of an upper limb muscle, in combination with one or more clinical measures of MS disease severity, motor impairment, cognitive impairment, or fatigue. Ten studies compared both TMS and clinical measures across MS and HC participants [8, 42, 63, 66, 71, 75, 77, 78, 80, 81]. Twenty studies examined correlations between TMS and clinical measures in MS participants [8, 42, 43, 54, 63, 65, 67–69, 71–75, 82–87].

#### 3.3.1. TMS Measures

**Figure 3:**
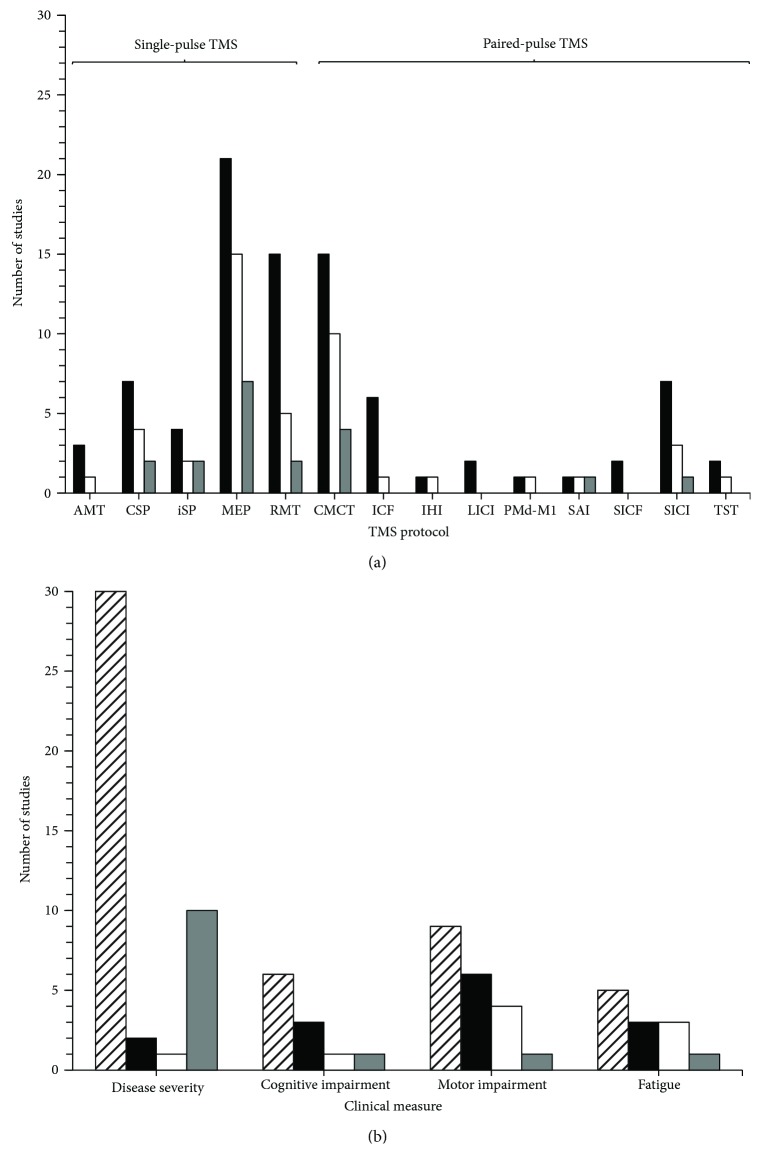
Summary of (a) transcranial magnetic stimulation (TMS) and (b) clinical outcome measures from studies reviewed. The horizontal axis indicates the outcome measure of interest, and the vertical axis represents number of studies utilizing each outcome measure. The black vertical bars (a and b) represent studies comparing both participants with multiple sclerosis (MS) and healthy control participants (HC). The white vertical bars (a and b) represent studies with statistically significant differences between MS and HC groups. The grey vertical bars (a and b) represent studies that found statistically significant correlations between TMS and clinical outcome measures in participants with MS. The hatched bar (b only) represents studies examining clinical outcome measures in MS participants alone. AMT: active motor threshold; CSP: cortical silent period; iSP: ipsilateral silent period; MEP: motor-evoked potential; RMT: resting motor threshold; CMCT: central motor conduction time; ICF: intracortical facilitation; IHI: interhemispheric inhibition; LICI: long-interval intracortical inhibition; PMd-M1: dorsal premotor cortex-primary motor cortex interactions; SAI: short-latency afferent inhibition; SICF: short-interval intracortical facilitation; SICI: short-interval intracortical inhibition; TST: triple stimulation technique.

Table 3 shows the brief descriptions of TMS protocols ([Supplementary-material supplementary-material-1] for in-depth descriptions), and Figure 3(a) illustrates the TMS findings. Twenty-seven studies employed single-pulse TMS paradigms [8, 42, 43, 54, 63–66, 68–76, 78–83, 85–88], and 23 studies utilized paired-pulse TMS protocols [42, 43, 54, 63, 65, 67–74, 77–79, 81–84, 86–88]. Overall, 25 studies found significant differences in TMS measures across MS and HC samples [8, 42, 43, 54, 63–77, 79, 81, 85–88].

*(1) Single-Pulse TMS*. *Motor-Evoked Potential (MEP)*. Twenty-one studies examined the aspects of the MEP, including amplitude (absolute values, *μ*V or mV [8, 42, 63, 66, 69–71, 73, 75, 79, 81, 82, 85], percentage of maximal compound motor unit action potential, %Mmax [43, 78, 87]), area [72, 75], linear slope of the recruitment curve [74, 78], latency (ms) [42, 63, 69, 71–75, 78, 79, 81, 82, 85–88], duration (ms) [74, 75], and number of turns [75] or abnormalities in the MEP waveform [68]. Among studies examining MEPs, nine found a significant decrease in MEP amplitude in persons with MS compared to HC participants [8, 42, 66, 69–71, 75, 85, 87]. Nine studies also observed a significant increase in MEP latency in MS versus HC [42, 63, 69, 71, 73–75, 79, 86]. Two studies found a significant increase in MEP duration in MS participants [74, 75]. One study each found persons with MS showed a significant decrease in the linear slope of the MEP recruitment curve [74] and exhibited a significantly higher MEP number of turns [75]. The MEP area was not significantly different across groups [72, 75]. Participants with progressive MS showed significantly smaller [42, 87], more latent MEPs [42, 86], with a greater number of waveform abnormalities than in persons with RRMS [68]. One study found persons with functional impairments because RRMS had smaller and more latent MEPs than persons with RRMS and preserved function [71]. Another study found more latent MEPs in the left versus right hemisphere of persons with RRMS [74].

*Resting Motor Threshold (RMT).* Fifteen studies reported on RMT, reported as percentage of maximum stimulator output (%MSO) [42, 43, 54, 64, 65, 70–75, 79–81, 87]. Five of these studies found increased RMT in participants with MS compared to HC participants [64, 72, 74, 75, 87]. One study noted greater RMT in persons with SPMS versus RRMS [87].

*Cortical Silent Period (CSP).* Seven articles investigated either CSP duration (ms) [42, 71, 74, 76, 78, 85, 87] or onset latency (ms) [74, 85]. Importantly, there were discrepancies in how the CSP was determined across studies. The CSP was defined as end of the MEP until the return of the EMG response from the contracted muscle [42, 71, 74], the downward deflection of the MEP until onset of EMG [76], or the beginning of the MEP until resumption of EMG [87]. Two studies did not describe methods to define the CSP [78, 85]. Two studies found that CSP duration was increased in participants with MS compared to HC [71, 85], whereas another study found decreased CSP duration in MS relative to HC participants [76]. RRMS participants with functional impairments had a prolonged CSP duration compared to those without functional impairments [71]. In addition, one study reported greater CSP onset latency in MS versus HC [74].

*Ipsilateral Silent Period (iSP).* Four studies utilized either iSP latency (ms) [54, 65, 73, 74], duration (ms) [54, 65, 73, 74], conduction time (difference between onset latencies MEP and EMG suppression, ms) [65, 73], or amplitude (mean EMG amplitude iSP/mean prestimulus EMG amplitude, %Pre-stim) [74]. iSP was defined as quantifiable suppression of the background EMG signal following TMS delivery over the ipsilateral hemisphere, until the return of normal background EMG activity [65, 73, 74]. One study did not report how the iSP was defined [54]. Three studies demonstrated increased iSP latency in MS compared to HC [54, 65, 73], two illustrated increased iSP conduction time in MS versus HC [65, 73], and one found increased iSP duration in MS as opposed to HC [65]. Conversely, one study found decreased iSP duration in MS participants compared to HC participants [73]. No between-group differences in iSP amplitude were reported [74].

*Active Motor Threshold (AMT).* Three studies investigated AMT (%MSO) [54, 70, 74]. One of these studies reported greater AMT in participants with MS versus the HC group [74].

*(2) Paired-Pulse TMS*. *Central Motor Conduction Time (CMCT)*. Fifteen articles reported on CMCT (ms) [43, 54, 65, 68–70, 72, 73, 77, 78, 82, 83, 86–88]. CMCT was determined by subtracting cervical spine corticomotor latency [43, 54, 65, 68–70, 73, 82, 83, 86], or the sum of F-wave and M-wave latencies [72, 78, 87, 88], from MEP latency. One study did not state the method used [77]. Ten of these studies reported increased CMCT in MS versus HC participants [54, 65, 68–70, 72, 73, 85–87]. Increases in CMCT were also reported in persons with progressive MS compared to RRMS [68, 86, 87]. In addition, CMCT was more prolonged MS participants' clinically more impaired upper limb compared to their less affected side [72].

*Short-Interval Intracortical Inhibition (SICI).* Seven studies assessed SICI, reported as the size of the conditioned MEP normalized to the unconditioned test MEP [42, 67, 70, 71, 74, 81, 87]. Three studies found significant reductions in SICI in MS versus HC participants [42, 81, 87]. Two of these revealed reduced SICI in persons with SPMS compared to both RRMS and HC [42, 87]; and one study found lower SICI in SPMS compared to PPMS as well [42]. Another study found that participants with RRMS and fatigue had lower SICI than persons with RRMS who were not experiencing fatigue [81].

*Intracortical Facilitation (ICF).* Six articles examined ICF, reported as the size of the conditioned MEP relative to the unconditioned test MEP [67, 70, 71, 74, 81, 87]. One of these studies found a significant increase in persons with SPMS compared to both HC participants and persons with RRMS [87].

*Triple Stimulation Technique (TST).* Two studies employed TST [84, 88]. In both studies, TST consisted of stimuli over M1, the ulnar nerve at the wrist, and the cervical spine [84, 88]. One revealed increased TST latency and TST latency variability in MS participants versus HC participants [88]. In the same study, TST latency variability was also greater in persons with PPMS compared to RRMS [88].

*Long-Interval Intracortical Inhibition (LICI).* Two records examined LICI, reported as the size of the conditioned MEP relative to the unconditioned test MEP [67, 71]. Neither of these articles reported a significant difference in LICI across MS and HC groups.

*Short-Interval Intracortical Facilitation (SICF).* Two articles used SICF, expressed as the size of the conditioned MEP relative to the unconditioned test MEP [67, 72]. No differences in SICF were found across groups in these studies [67, 72].

*Interhemispheric Inhibition (IHI).* One article assessed IHI, reported as the size of the conditioned MEP relative to the unconditioned test MEP [77]. This study found a significant decrease in short-interval (SIHI), but not long-interval (LIHI), interhemispheric inhibition in MS versus HC participants [77].

*Dorsal Premotor-Primary Motor Cortex Interactions (PMd-M1).* One study examined PMd-M1 interactions across participant groups, reported as the size of the conditioned MEP relative to the unconditioned test MEP [67]. The authors observed reduced PMd-M1 facilitation in RRMS compared to HC participants [67]. As well, PMd-M1 inhibition was greater in in RRMS participants with disability versus HC [67].

*Short-Latency Afferent Inhibition (SAI).* SAI was explored in one study, reported as the size of the conditioned MEP relative to the unconditioned test MEP [43]. In this study, SAI was found to be lower in MS compared to HC participants [43].

#### 3.3.2. Clinical Measures

Brief descriptions of clinical measures employed in the studies can be found in Table 4 ([Supplementary-material supplementary-material-1] for in-depth descriptions). Results pertaining to clinical measures can be found in Figure 3(b). Twenty studies explored correlations between clinical measures and TMS outcomes in MS participants [8, 42, 43, 54, 63, 65, 67–69, 71–75, 82–87]; 11 of which found statistically significant results [43, 54, 63, 65, 69, 71, 72, 74, 85–87].

*(1) Disease Severity*. EDSS was positively related to CMCT (three studies) [65, 72, 87], MEP latency (three studies) [63, 85, 86], RMT (two studies) [74, 87], iSP latency (two studies) [54, 65] and duration (one study) [65], and CSP duration [71]. EDSS was negatively related to MEP amplitude (three studies) [69, 85, 87], recruitment curve slope (one study), [74], and SICI (one study) [87].

Two studies found significant relationships between Kurtzke's Functional Systems Scores (Kurtzke FSS) and TMS measures [85, 86]. One study each found a positive correlation between the Kurtzke FSS Pyramidal Domain and MEP amplitude [85] and latency [86] and CMCT [85]. One study each also found a positive relationship between the Kurtzke FSS Cerebellar Domain and MEP amplitude [85] and latency [86] and CSP duration [85]. One study found a positive correlation between the Kurtzke FSS Sensory Domain and MEP latency [85]. In addition, one study reported a positive relationship between MSFC and MEP latency [71].

*(2) Cognitive Impairment*. One study observed significant relationships between each of the Selective Reminding Test Long-Term Storage (SRT-LTS), Consistent Long-Term Retrieval (SRT-CTLR), and Delayed Recall (SRT-DR) domains and SAI [43].

*(3) Motor Impairment*. One study found a positive correlation between 9HPT time to completion and both MEP latency and CSP duration [71].

*(4) Fatigue*. One record reported a positive relationship between FSS and MEP latency [86].

*(5) Neuroimaging*. As an adjunct to the *a priori* clinical outcomes of interest, 10 studies explored relationships between TMS findings and MRI-based neuroimaging outcomes. Of these studies, six found statistically significant relationships between TMS and MRI findings in persons with MS.

*Structural MRI.* Two studies found that lesion load was significantly related to CSP duration [42] and CMCT [87]. Using diffusion tensor imaging, one study found white matter microstructural characteristics of the corpus callosum to be correlated negatively with iSP latency (radial diffusivity and mean diffusivity) [54], while in another study a significant positive relationship between SIHI and fractional anisotropy found in HCs was absent in MS participants [77]. In terms of brain volumes, one study found significant negative correlations between iSP latency and corpus callosum volume and area, as well as normalized brain parenchymal volume, normalized normal-appearing white matter volume, and normalized gray matter volume; another study found that in MS participants with corpus callosum atrophy, there were greater abnormalities in MEP amplitude and latency and CMCT compared to both HCs and MS participants without corpus callosum atrophy [69].

*Functional MRI*. One study observed that the activation of the primary motor cortex ipsilateral to the hand performing rapid finger movements was significantly correlated with iSP duration [73].

## 4. Discussion

There is a growing imperative to establish viable biomarkers of disease status in MS [2, 24]. Clinical presentation is highly variable, and disease progression is unpredictable [11, 13, 15, 16]; differentiation between MS subtypes is based almost solely on clinical features with few tools to guide treatments [26, 27], and changes in myelination can be observed early in the disease when affected individuals experience little or no impairment [2, 18, 19]. However, without early intervention, many individuals will experience permanent disability as the disease progresses [3, 11, 14, 16, 23]. Biomarkers are used in the diagnosis, characterization, prognostication, and surveillance of disease throughout its natural history and in response to therapy [25]. To advance the role of TMS as a biomarker in clinical assessments of MS, the viability of this technique must be better established through the interrogation of study quality.

In the present review, we examined the utility of TMS as a biomarker in cross-sectional comparisons of persons with MS and HC participants. Other reviews have discussed the use of TMS in longitudinal studies of MS [24, 34]. Currently, we aimed to (i) systematically assess the methodological quality and risk of bias in studies of TMS and clinical outcomes of disease severity, cognitive impairment, motor impairment, or fatigue in MS and (ii) synthesize the findings of these studies, including relationships between TMS and clinical outcomes. Thus, we endeavoured to explore the viability of TMS for diagnostic and characterization purposes in MS [25]. While similar efforts have been made for other neurological disorders [45, 46], this is the first such review in MS. After reviewing 1130 records, 30 studies were finally retained for risk of bias assessment and data extraction. Here, we will outline major areas of concern gleaned from quality assessment, highlight which TMS methodologies may most promising for future work, and identify suggestions for future research.

### 4.1. Risk of Bias and Quality Assessment

Based on our systematic risk of bias and quality assessment, all 30 studies were deemed to be at a high risk of bias. The foremost area of concern was sample size justification. Only two studies conducted sample size calculation to inform the size of their participant sample [63, 64], despite most studies employing small sample sizes. No study provided estimates of effect size. Given only 10 studies [42, 65, 67–71, 75, 85, 87] examined large samples of MS participants (*n* ≥ 30) [89], the findings of small studies could be underpowered or have insufficient effect sizes [90]. To establish the appropriateness of TMS for clinical use, it is important to determine where marginal differences in outcomes between MS and HC samples would be statistically significant, especially in when considering persons with low clinical impairment, who may have preclinical changes in motor system integrity [2, 18, 19]. It is thus critical for future work to be informed by sample size calculation and to report estimates of effect sizes [90].

The second most significant source of potential bias was related to experimenter blinding. Fewer than half (11/30) of the studies reviewed employed rater blinding during at least one study component [8, 42, 43, 54, 65–71]. Blinding is an integral part of any evaluation of biological markers or diagnostic tests [91], given the need to remove bias in favour of the investigation of interest. Consequently, experimenter blinding at all possible stages of research should be prioritized in future work [91, 92].

Thirdly, just over half (18/30) of studies adequately accounted for key potential confounding variables, by controlling for or acknowledging at least 19 sources of possible confounding [42, 43, 63–67, 69–79]. Areas of principal concern include the history of specific repetitive motor activity (0 studies), participants' level of attention during TMS testing (one study [64]), and nonprescription drug or supplement usage by participants (one study [64]), where virtually no study accounted for these potential confounds. Overall, there were 13 key potential confounding factors not considered by at least half of the studies reviewed. This suggests that studies require more rigorous experimental design and planning, methodological and statistical controls, and reporting of results. Indeed, as Chipchase et al. [40] highlight, there is considerable inter- and intraindividual variability in TMS findings and a strong potential for methodological and physiological differences to influence TMS responses. This is particularly important in the clinical evaluation of MS, where significant variability in clinical presentation and unpredictability of clinical course characterize the disease phenotype [11, 13, 15, 16].

Taken together, the above outcomes suggest that further research is required to assert the viability of TMS as a clinical marker of MS disease status. Other critical reviews in stroke [45] and hereditary ataxias [46] have made similar conclusions. As such, the present review may help serve as a template to inform future experimental design in MS.

### 4.2. Data Synthesis

Despite concerns surrounding overall study quality, our findings highlight some compelling TMS methods that should be investigated further in clinical studies. TMS may have the most value in characterizing axonal conduction [24, 34, 35, 37] and neurotransmitter signaling [2], both of which are disturbed in MS.

Demyelination of fast-conducting corticospinal motor neurons slows corticomotor conduction times in persons with MS, revealed by changes in CMCT and MEP latency [37, 93]. As well, asynchronous activation of corticospinal neurons results in phase cancellation, evidenced by changes in MEP and CMCT [37, 93]. CMCT [54, 65, 68–70, 72, 73, 85–87], MEP amplitude [8, 42, 66, 69–71, 75, 85, 87], and MEP latency [42, 63, 69, 71, 73–75, 79, 86] were the most widely reported outcomes with positive findings across MS and HC groups. CMCT [65, 72, 85, 87], MEP amplitude [69, 85, 87], and MEP latency [63, 71, 85, 86] also showed small to moderate correlations with measures of disease severity. MEP latency was also related to motor impairment [71] and fatigue [86]. Studies also found significant differences in CMCT [68, 86, 87], MEP amplitude [42, 87], and MEP latency [42, 86] in persons with progressive MS versus RRMS. Others have suggested that measures of corticomotor latency have the greatest clinical utility among TMS techniques used in MS research [24, 34, 35, 37] and may be particularly useful for identifying clinically silent CNS lesions [94]. As such, MEP latency and CMCT may be among the more useful clinical functions of TMS in assessments of persons with MS. Additionally, one study found lower SAI in MS compared to HC participants [43], with SAI being moderately correlated with cognitive impairment [43]. SAI was the only TMS measure related to cognition [43]. However, more studies are required to elucidate the utility of SAI.

Glutamate-mediated excitotoxicity [2] is thought to disrupt long-term potentiation (LTP) [95–97] in MS. MEPs and motor thresholds (AMT and RMT) are reportedly influenced by glutamate [44, 98]. As indicated above, MEP characteristics were some of the most robust outcome measures to distinguish MS from HC participants, characterize the disease, and relate to clinical outcomes. Several studies found increased RMT and AMT in MS versus HC participants [64, 72, 74, 75, 87], while one study found an increase in RMT in progressive MS compared to RRMS [87]. However, no studies reported correlations between motor thresholds and clinical outcomes. The findings from these studies suggest that motor thresholds and may have less clinical utility in MS evaluation, in contrast with the greater evidence in favour of MEP characteristics. Recent work suggests that better control of confounding variables can enhance the clinical utility of motor thresholds, for instance, by accounting for interhemispheric differences in corticospinal excitability [99]. This evidence is well supported by our observation that many studies reviewed did not adequately control for key potential confounding variables.

TMS findings in MS could also be related to changes in GABA-ergic or cholinergic signaling, both of which are disturbed throughout the disease [98, 100–102]; however, given much of this evidence is gleaned from animal models or indirect findings, this discussion is highly speculative. Disruption in GABA-ergic transmission accompanies glutamate-mediated excitotoxicity [101, 102] and may be related to motor features in MS [102]. In animal research, the administration of valproic acid and phenobarbitone, drugs acting on GABA_A_ receptors, was found to improve clinical status and inhibits glutamate-mediated excitotoxicity [101]. Pharmaceutical treatment of humans with intrathecal infusion of baclofen, a GABA_B_ agonist, reduces spasticity with an accompanying increase in CSP latency and duration [103]. Blocking acetylcholine activity is associated with reduced LTP [104], while increasing synaptic availability of this neurotransmitter is been linked to improved myelination and clinical symptoms in MS [100]. Animal model studies of MS involving nicotine—a potent modulator of nicotinic acetylcholine receptors expressed in immune cells and glial cells alike—have shown reductions in demyelination and neuroinflammation, as well as improvements in clinical status, following nicotine administration [105]. A recent review of human studies suggested a link between reduced acetylcholine expression and potency and increased neuroinflammation and cognitive deficits in persons with MS [106]. In line with this proposed relationship, Cucurachi et al. [43] found that reductions in SAI were significantly related to cognitive dysfunction in persons with MS but were significantly improved following the administration of rivastigmine, a cholinesterase inhibitor that prevents synaptic breakdown of acetylcholine. Indeed, SAI is thought to be related to both acetylcholine and GABA_A_ receptor activity [98], and is a compelling technique for use in MS; however, more evidence is required in this field. GABA_A_-ergic intracortical networks have been deemed important in SICI [98, 107], and some studies found reduced SICI in MS versus HC participants [42, 81, 87], as well as SPMS compared to both RRMS [42, 87] and PPMS [42]. SICI was also moderately correlated with disease severity [87]. Additionally, GABA_B_-ergic connections are thought to underlie CSP [35, 44, 98, 108, 109]. CSP duration was related to both disease severity [71, 85] and motor impairment [71] in persons with MS. However, findings related to CSP were variable across studies, reporting both increases [71, 74, 85] and decreases [76] in MS versus HC participants. It is important to note that studies used various definitions for the CSP. Thus, it is critical for future work to standardize and report approaches to determining CSP.

### 4.3. Future Directions

Given the argument for compelling TMS methods for future investigation in MS, it is worth noting putative applications of TMS in the clinical approach to MS, once methodological limitations such as those from the cited studies have been overcome. As mentioned previously, recommended uses of biomarkers include disease diagnosis, assessment, prognostication, and surveillance [25] and TMS has the potential to be a valid and reliable biomarker. Since the present review focused on cross-sectional comparisons of participants, the most apparent clinical applications of TMS presented here are cross-sectional in nature. For instance, damage to gray matter and white matter occurs prior to the emergence of obvious clinical sequelae [2, 18–22]; one appropriate approach for TMS may be as a screening tool for MS. Indeed, Tataroglu et al. [85] found that sensitivity and specificity of CMCT, CSP, and MEPs were up to 89.7% and 96.7%, respectively, suggesting that TMS may be useful to rule out MS in a healthy person under investigation [110]. These values are similar for other biomarkers under development such as NfL (sensitivity: 89.5%, specificity: 95.4%) [32], while other modalities such as MRI are suggested to lack the sensitivity and specificity required for a valid screening tool [29]. Others have likewise suggested that TMS is a useful candidate screening tool for MS, with a sensitivity up to 93% [24, 35].

Additionally, TMS may have utility for discerning between RRMS and progressive MS subtypes or progression from RRMS to SPMS. Presently, clinical presentation in MS is highly variable and disease progression is unpredictable [11, 13, 15–17], while differentiation between RRMS and progressive MS is largely based on clinical observations [26, 27]. Nevertheless, the current studies highlight that TMS techniques including MEP characteristics [42, 68, 86, 87], RMT [87], CMCT [68, 86, 87], SICI [42, 87], ICF [87], and TST [88] could be used to discern progressive MS from RRMS.

## 5. Conclusions

Due in part to a relative paucity of biological markers for MS disease status, some authors have promoted the use of TMS in clinical assessments of MS. However, our findings suggest that existing TMS research in the clinical study of MS is at a notable risk of potential bias and further research is required to assert the viability of TMS as a clinical marker of MS disease status. While we believe that the evidence is insufficient to support widespread use of TMS in clinical assessments of MS, our findings may help inform future experiments that will further support the clinical value of TMS. Overall, we suggest that MEP (particularly latency) and CMCT have the most evidence for use as biomarkers in research and the clinical approach to MS. Other techniques such as SAI, SICI, and CSP may have promise but require more evidence. In the future, TMS appears to have the greatest potential for use as a screening tool or to differentiate between disease subtypes or progression. In addition, current research is exploring the plausibility of TMS as a therapeutic modality for MS [111]; much of this evidence to date has been summarized and evaluated in another systematic review elsewhere [112].

## Figures and Tables

**Table 1:** Criteria used for risk of bias assessment [53].

Criterion description	
(1)	Was the research question or objective in this paper clearly stated?
(2)	Was the study population clearly specified and defined?
(3)	Was the participation rate of eligible persons at least 50%?
(4)	(a) Were all the subjects selected or recruited from the same or similar populations (including the same time period)? (b) Were inclusion and exclusion criteria for being in the study prespecified and applied uniformly to all participants?
(5)	Was a sample size justification, power description, or variance and effect estimates provided?
(6)	For the analyses in this paper, were the exposure(s) of interest measured prior to the outcome(s) being measured?^∗^
(7)	Was the timeframe sufficient so that one could reasonably expect to see an association between exposure and outcome if it existed? ^∗^
(8)	For exposures that can vary in amount or level, did the study examine different levels of the exposure as related to the outcome (e.g., categories of exposure or exposure measured as continuous variable)?
(9)	Were the exposure measures (independent variables) clearly defined, valid, reliable, and implemented consistently across all study participants?
(10)	Was the exposure(s) assessed more than once over time?^∗^
(11)	Were the outcome measures (dependent variables) clearly defined, valid, reliable, and implemented consistently across all study participants?
(12)	Were the outcome assessors blinded to the exposure status of participants?
(13)	Was loss to follow-up after baseline 20% or less?^∗^
(14)	Were key potential confounding variables measured and adjusted statistically for their impact on the relationship between exposure(s) and outcome(s)?^ǂ^

^∗^Because we examined studies using cross-sectional comparisons where exposure status was apparent (i.e., MS vs. HC), criteria 6, 7, 10, and 13 (loss to follow-up) were not evaluated. ^ǂ^Key confounding variables are listed in Table 2.

**Table 2:** Key confounding variables evaluated in risk of bias assessment [40, 54–61, 78, 113].

Key confounding variable description
(i) Age of participants.
(ii) Sex of participants.
(iii) Handedness of participants.
(iv) Participants prescribed medication.
(v) Use of CNS active drugs (e.g., anticonvulsants).
(vi) Presence of neurological/psychiatric disorders when studying healthy participants.
(vii) Any medical conditions.
(viii) History of specific repetitive motor activity.
(ix) Position and contact of EMG electrodes.
(x) Amount of relaxation/contraction of target muscles.
(xi) Prior motor activity of the muscle to be tested.
(xii) Level of relaxation of muscles other than those being tested.^∗^
(xiii) Coil type (i.e., size and geometry).
(xiv) Coil orientation.
(xv) Direction of induced current in the brain.
(xvi) Coil location and stability (with or without a neuronavigation system).
(xvii) Type of a stimulator used (e.g., brand).
(xviii) Stimulation intensity.
(xix) Pulse shape (i.e., monophasic or biphasic).
(xx) Determination of optimal hotspot.
(xxi) The time between MEP trials.
(xxii) Time between days of testing.
(xxiii) Participant attention (i.e., level of arousal) during testing.
(xxiv) Method for determining threshold (i.e., active/resting).
(xxv) Number of MEP measures made.
(xxvi) Paired-pulse only: intensity of test pulse.^ǂ^
(xxvii) Paired-pulse only: intensity of conditioning pulse.^ǂ^
(xxviii) Paired-pulse only: interstimulus interval.^ǂ^
(xxix) Method for determining MEP size during analysis.
(xxx) Size of unconditioned MEP.
(xxxi) Disease duration in MS.
(xxxii) Disease severity in MS.
(xxxiii) MS subtype (i.e., relapsing-remitting and progressive).
(xxxiv) Participants experiencing a relapse in MS.
(xxxv) Participants receiving corticosteroid treatment for MS.
(xxxvi) Participants undergoing immunomodulatory treatment for MS.
(xxxvii) Participants taking nonprescription or recreational drugs (e.g., caffeine and nicotine).
(xxxviii) Room or skin temperature reported.

CNS: central nervous system; EMG: electromyography; MEP: motor-evoked potential; MS: multiple sclerosis. ^∗^Criterion (xxii) was disregarded because we did not examine longitudinal studies. ^ǂ^Criteria (xxvi), (xxvii), and (xxviii) were marked NA, when studies did not utilize paired-pulse TMS measures.

**Table 3:** Transcranial magnetic stimulation (TMS) measures employed in studies.

TMS protocol	Stimulation characteristics	Neural mechanisms	Studies utilizing
Single-pulse TMS			
Active motor threshold (AMT)	% MSO to elicit a 100-200 *μ*V MEP in ≥5/10 trials during a tonic contraction of the target muscle [37, 44, 114, 115].	Corticospinal excitability; influenced by Glu [33, 98].	[54, 70, 74]
Cortical silent period (CSP)	Reduced background EMG following MEP when TMS is delivered during a tonic contraction of the target muscle contralateral to M1 [37, 44, 74, 116–119].	Spinal and cortical inhibition; influenced by GABA_B_Rs [35, 44, 98, 108, 109].	[42, 71, 74, 76, 78, 85, 87]
Ipsilateral silent period (iSP)	Reduced background EMG when TMS is delivered during a tonic contraction of the target muscle ipsilateral to M1 [65, 74, 118, 120–123].	Transcallosal inhibition; influenced by GABA_B_Rs [33, 123–125].	[54, 65, 73, 74]
Motor-evoked potential (MEP)	Deflection in the EMG trace of the target muscle following the delivery of a TMS pulse over the M1 [44, 126, 127].	Amplitude reflects corticospinal excitability; latency reflects corticomotor latency; influenced by Glu, GABA, 5-HT, and NE [37, 44, 68, 107, 126–129].	[8, 42, 43, 63, 66, 68–75, 78, 79, 81, 82, 85–88]
Resting motor threshold (RMT)	% MSO to elicit a 50 *μ*V MEP in ≥5/10 trials in a resting target muscle [44, 114].	Corticospinal excitability; influenced by Glu [33, 98, 115, 127].	[42, 43, 54, 64, 65, 70–75, 79–81, 87]

Paired-pulse TMS			
Central motor conduction time (CMCT)	Difference between spinal cord-/brainstem-to-muscle MEP latency and M1-to-muscle MEP latency [35, 37, 44, 114, 130–133].	Corticomotor latency [33, 35, 37, 93].	[43, 54, 65, 68–70, 72, 73, 77–79, 82, 83, 86–88]
Dorsal premotor-primary motor cortex interaction (PMd-M1)	Sub-/suprathreshold CS over PMd and suprathreshold TS over M1 [67, 134, 135].	Cortical inhibition/facilitation [67, 134].	[67]
Interhemispheric inhibition (IHI)	Sub-/suprathreshold CS and suprathreshold TS over homologous M1 representations [35, 44, 77, 122, 135–137].	Cortical inhibition/facilitation; influenced by GABA_B_Rs [122, 135–138].	[77]
Intracortical facilitation (ICF)	Subthreshold CS followed 10-15 ms later by a suprathreshold TS [33, 35, 44, 139].	Cortical facilitation; influenced by Glu [35, 98, 107, 140].	[42, 67, 70, 74, 81, 87]
Long-interval intracortical inhibition (LICI)	Suprathreshold CS and TS separated by 50-200 ms [44, 141–143].	Intracortical inhibition; influenced by GABA_B_Rs [98, 144, 145].	[42, 67]
Short-interval intracortical facilitation (SICF)	Sub-/suprathreshold CS and TS separated by 1.1-4.5 ms [44, 67, 121, 146–149].	Cortical facilitation; influenced by GABA_A_Rs [35, 37, 44, 98, 107, 146, 147].	[67, 72]
Short-interval intracortical inhibition (SICI)	Subthreshold CS and suprathreshold TS separated by 1-5 ms [37, 114, 139, 143, 150].	Intracortical inhibition; influenced by GABA, GABA_A_Rs [98, 107, 144, 151, 152].	[42, 67, 70, 71, 74, 81, 87]
Short-latency afferent inhibition (SAI)	Electrical CS over median nerve followed by a suprathreshold TMS TS over 20-25 ms later [44, 153–155].	Sensorimotor integration; influenced by Ach and GABA_A_Rs [33, 98, 153, 155].	[43]
Triple stimulation technique (TST)	Suprathreshold TMS delivered over M1 and electrical stimulation over proximal and distal ends of the peripheral nerve supplying the target muscle [35, 156, 157].	Corticospinal conduction [37, 88, 157, 158].	[84, 88]

MSO: maximal stimulator output; Glu: glutamate; EMG: electromyogram; MEP: motor-evoked potential; M1: primary motor cortex; GABA_B_R: *γ*-aminobutyric acid receptor B; GABA: *γ*-aminobutyric acid; 5-HT: serotonin; NE: norepinephrine; CS: conditioning stimulus; TS: test stimulus; GABA_A_R: *γ*-aminobutyric acid receptor A; Ach: acetylcholine.

**Table 4:** Clinical measures employed in studies.

Clinical measure	Test characteristics	Studies utilizing
Disease severity		
Expanded Disability Status Scale (EDSS)	Ordinal scale based on observations concerning gait and use of assistive devices. Rated from 0.0 to 10.0, in increments of 0.5, where 0.0 indicates no disability, 1.0-4.5 describes persons who can walk without mobility aids, 5.0-9.5 refers to impairments in walking, ranging from being able to walk 200 m without aid (5.0) to being confined to bed and unable to communicate or swallow (9.5), and 10.0 indicates the person has died [51, 55, 67].	[8, 42, 43, 54, 63–68, 70–72, 74–79, 83–88]
Kurtzke's Functional Systems Scores (Kurtzke FSS)	Set of eight ordinal subscales based on the standard neurological examination. Each is rated from 0 to 9 in discrete increments of 1, where greater score denotes more severe disability. Subscales include pyramidal function, cerebellar function, brainstem function, sensory function, bowel and bladder function, visual function, cerebral/mental function, and other features noted by the examiner. Scores can be reported separately or as a composite [159].	[68, 85, 86]
Multiple Sclerosis Functional Composite (MSFC)	Battery containing the Timed 25-foot Walk (T25FW), Nine-hole Peg Test (9HPT), and Paced Auditory Serial Addition Test-3 seconds (PASAT3) to assess leg function/ambulation (T25FW), arm/hand function (9HPT), and cognitive function (PASAT3), respectively. Each item can be scored separately or combined [160].	[8, 42, 54, 71]

Cognitive impairment		
Brief Repeatable Battery (BRB)	Includes elements of the selective reminding test (SRT) (verbal memory), Spatial Recall Test (SPART) (visual memory), Symbol Digit Modalities Test (SDMT) (attention, visual precision search, processing speed, and executive functions), paced auditory serial addition test (PASAT) (maintenance of attention, processing speed, and working memory), world list generation (WLG) (associative verbal fluency), and Stroop test (ST) (selective attention). Subscales can be scored independently, collapsed across specific cognitive domains (i.e., verbal memory and visual memory), or combined [54, 161].	[54]
California Verbal Learning Test (VLGT)	Test of episodic verbal learning and memory. A list of nouns is read aloud over several trials, after each of which the participant attempts to recall as many nouns as possible. Participants are also provided with an interference list of words with similar meaning. Recall and recognition of the original list are tested at different intervals. A learning curve with learning parameters, response errors, and interference effects is used for scoring [162–164].	[80]
Digit span	Test of short-term verbal memory. Sequences of digits are presented in forward and reverse order, and the participant recalls the sequences. Two trials are presented at each sequence length, beginning with two digits, until either the participant fails to recall the trial or the maximal span length is reached (nine forward and eight backward). The total number of lists recalled correctly is combined across forward span and backward spans to give a total correct score [165–167].	[80]
Frontal Assessment Battery (FAB)	Test of frontal lobe dysfunction. Utilizes six subscales that examine conceptualization (similarities test), mental flexibility (verbal fluency test), motor programming (Luria motor sequences), sensitivity to interference (conflicting instructions), inhibitory control (go-no-go test), and environmental autonomy (prehension behaviour). Each subscale is rated from 0 to 3, and the sum of the scores is interpreted; 18 is the maximum (best) score, and <12 indicates cognitive impairment [168].	[8]
Letter Digit Substitution Test (LDST)	Test of information processing speed and visual or auditory memory. Administered in a visual or auditory format, digits 1 to 9 are associated with a corresponding letter. Participants must replace randomized letters with the appropriate digit as quickly as possible. Scoring is based on the number of correct letter-digit substitutions made in 60 seconds [169, 170].	[80]
Location Learning Test (LLT)	Test of visuospatial learning and memory. Participants are shown an array of images several times for 30 seconds at a time. After each presentation and 15 minutes after the last presentation, participants must relocate the images to their correct position on an empty grid. For every trial, a displacement score is measured consisting of the sum of the errors made for each object placement on that trial, a total displacement score combines the individual displacement scores from the learning trials, a learning index represents the relative difference in performance between trials, and a delayed recall score considers the difference between last trial and delayed trial [171, 172].	[80]
Letter-Number Sequencing (LNS)	Test of auditory or visual working memory and attention. In either auditory or visual form, the participant is presented a series of letters and digits in a nonsystematic order. Following the presentation, the participant must report back the stimuli, with the letters in alphabetical order and the digits in ascending order. Scoring is based on correctness of responses [165, 173, 174].	[80]
Mini Mental State Exam (MMSE)	Thirty-point questionnaire examining aspects of cognitive function including registration, attention, calculation, recall, language, ability to follow simple commands, and orientation. Scoring is relative to age- and education-based norms [175, 176].	[83]
N-back	Test of processing speed and working memory. Computer task whereby participants press one of two buttons, denoting target and nontarget, in response to a target (letter) that matches a stimulus presented zero, one, two, or three stimuli previously. Scoring is based on reaction time and correctness of responses in each condition [177, 178].	[80]
Paced Auditory Serial Addition Test (PASAT-2/PASAT-3)	Test of processing speed and working memory. A series of digits is presented, either visually or aurally, and the two most recent digits must be summed. An interval of 2 (PASAT-2) or 3 seconds (PASAT-3) separates each digit. Scoring is based on the number of correct responses for each trial or the total number of correct responses over all trials. The PASAT is part of the MSFC and BRB [160, 161, 179].	[8, 43, 54, 71]
Posner test	Test of attention. Computer task involving responding to visual stimuli presented in one of two possible locations on the computer screen. Prior to the stimulus, a visual cue directs the participant's attention either to the correct location (valid cue) or an incorrect location (invalid cue). There are a proportionate number of valid and invalid cues and noncued stimuli, which are randomly interspersed. Performance is based on correct responses and reaction time and can be compared across cue conditions [8, 180].	[8]
Selective Reminding Test- (SRT-) LTS/CLTR/D	Test of verbal memory and learning. The participant hears a list of unrelated words and must recall as many words as possible. Every subsequent trial involves the administrator reminding the participant only of those words the participant did not recall on the previous trial. Trials of recall and selective reminding continue until the participant can correctly recall all words on three consecutive trials or until all trials have been completed. Scores are provided for words recalled from long-term storage (SRT-LTS), consistently from long-term retrieval (SRT-CLTR), and delayed recall (SRT-D). The SRT is part of the BRB [161].	[8, 43, 54]
Spatial Recall Test (SPART/SPART-D)	Test of visuospatial learning. A 6 × 6 checkerboard displaying a pattern of checkers is placed in front of the participant for 10 seconds. The participant tries to reproduce the pattern. This occurs for multiple trials, plus a 15-minute delayed-recall trial. Scoring is based on the number of correctly placed checkers over the first trials (SPART), as well as during the delayed-recall trial (SPART-D). The SPART is part of the BRB [161, 181].	[8, 43, 54]
Stroop test	Test of selective attention. Participants are instructed to read aloud a list of colour names as quickly as possible, leaving no errors uncorrected. The task utilizes five words (red, blue, green, brown, and purple) and their matching ink colours. Each ink colour appears twice in each row and column on 10‐word × 10‐word card. The task examines the effect of incompatible ink colour on reading words aloud and measures response time. The Stroop test is a component of the BRB [54, 161, 182].	[8]
Symbol Digit Modalities Test (SDMT)	Test of attention, visual precision search, processing speed, and executive function. participant is given 90 seconds to pair specific numbers with given geometric figures, based on a reference key provided by the experimenter. Scoring is based on a predetermined scoring form. The SDMT is a component of the BRB [161, 183, 184].	[8, 43, 54]
Word List Generation (WLG)	Test of verbal fluency, including category fluency (ability to list objects in different categories) and letter fluency (ability to list different words beginning with the same letter), semantic memory, and retrieval from long-term memory storage. Participants are asked to say as many different words as possible that begin with a specific letter (letter fluency) in 60 seconds. Participants cannot say proper nouns nor variations of the same word root. Next, participants must say as many words as possible from a specific category (category fluency) in 60 seconds. This test is part of the BRB [161, 185–187].	[80]

Motor impairment		
Grip strength	Used to describe hand function and to index overall body strength. Using a standard protocol, a handgrip dynamometer is used to measure handgrip strength. Results can be compared to norms, between individuals, or across limbs. Grip strength can also be measured as pinch grip strength or as a maximum voluntary isometric contraction (MVC) [188].	[71, 75, 81]
Medical Research Council (MRC) Strength Scale	Ordinal scale used to examine muscle strength, based on a standard neurological examination. The experimenter grades muscle strength on a scale of 0-5, relative to the maximum expected strength. A score of 0 indicates no contraction of the muscle, while 5 indicates normal strength [82, 189, 190].	[69, 82]
Modified Ashworth Scale (MAS)	Ordinal scale used to assess spasticity, based on a standard neurological examination. Uses discrete ratings of 1 and is scored from 0 to 4; 0 reflects normal tone3z and 4 indicates that the tested muscle is rigid during flexion or extension. The examiner passively flexes and extends joints of interest, providing a rating for each [189, 191].	[72]
Nine-hole Peg Test (9HPT)	Used to examine finger dexterity. The participant sits at a table with a small, shallow container holding nine pegs and a block containing nine empty holes. On a start command, the participant picks up and places each of the nine pegs in the nine holes as fast as possible, one at a time. The participant then removes them as quickly as possible, placing them into their container. The total time to complete the task is recorded. Two consecutive trials with the dominant hand are immediately followed by two consecutive trials with the nondominant hand. Both trials for each hand are averaged and reported separately. The 9HPT is part of the MSFC [160].	[42, 63, 71, 77]
Reflexes	Ordinal scale used to assess the presence or severity of “upper” versus “lower” motor neuron lesions, based on a standard neurological examination. A tendon is tapped briskly by a reflex hammer, and the resultant muscle contraction is given a score of 0-4, where 0 reflexes abnormal hyporeflexia, 2 is normal, and 4 denotes abnormal hyperreflexia [82, 189, 192].	[69, 82]
Timed 25-foot Walk (T25FW)	Used to assess walking performance and lower extremity function. The participant is instructed to walk as fast and safely as possible across a marked 25-foot linear course, using an assistive device if necessary. The participant is timed walking the course twice, and the two trials are averaged. Scoring is expressed as time or speed or as part of the ambulatory index, a 10-point scale that assesses mobility based on time and degree of assistance required during the T25FW. The T25FW is a component of the MSFC [160, 193, 194].	[71]

Fatigue		
Fatigue Impact Scale (FIS)	Self-report measure used to examine participants' perceptions of how fatigue impacts their quality of life. The scale is comprised of 40 items that are scored from 0 (no problem) to 4 (extreme problem), providing a total composite score of 0-160, and contains subdomains that reflect perceived impact on cognitive (concentration, memory, thinking, and organization of thoughts), physical (motivation, effort, stamina, and coordination), and psychosocial functioning (isolation, emotions, workload, and coping) (10 items/40 points each) [195].	[78]
Fatigue Severity Scale (FSS)	Self-report measure that uses a series of 7-point scales to examine the severity and impact of subjective feelings of fatigue. In response to each of the nine statements provided, a rating of 1 indicates strong disagreement while 7 refers to strong agreement. A total score < 36 indicates that the individual may not be suffering fatigue, whereas >36 suggests that one may be experiencing fatigue and should seek medical counsel [196].	[66, 75]
Modified Fatigue Impairment Scale (MFIS)	Abbreviated version of the FIS that has been adapted for persons with MS. This scale contains cognitive (9 items/36 points), physical (10 items/40 points), and psychosocial (2 items/8 points) subscales; however, this test only contains 21 items and can be rated out of a total 0-84 points [195, 197].	[8, 87]

## Supplementary Materials

Supplementary Materials Figure S1: key potential confounding variable findings. In-depth descriptions of the TMS and clinical outcomes employed in reviewed studies can be found in Tables S1 and S2, respectively. See Tables S3 and S4 for results pertaining to article screening and data extraction, respectively. Original review protocol can be found in Supplemental Methods 1-2.
