# Supplementary material for: Transcranial Magnetic Stimulation as a Potential Biomarker in Multiple Sclerosis: A Systematic Review with Recommendations for Future Research
Source: Neural Plast. 2019 Sep 16;2019:6430596. doi: 10.1155/2019/6430596 (PMC6766108; doi:10.1155/2019/6430596)
Supplement: Supplementary Materials — Figure S1: key potential confounding variable findings. In-depth descriptions of the TMS and clinical outcomes employed in reviewed studies can be found in Tables S1 and S2, respectively. See Tables S3 and S4 for results pertaining to article screening and data extraction, respectively. Original review protocol can be found in Supplemental Methods 1-2. [file 6430596.f1.zip › Supplemental Methods 1 - Review Protocol.docx]

**Review Information (Follows PORSPERO Online Guide Format Available** [**Here**](https://www.crd.york.ac.uk/PROSPERO/documents/Registering%20a%20review%20on%20PROSPERO.pdf)**)**

**Review Title and Timescale**

**Title:** Using transcranial magnetic stimulation to detect cognitive, motor, and fatigue outcomes

in multiple sclerosis.

**Anticipated start date:** September 15, 2017.

**Anticipated completion date:** February 28, 2019

**Stage of review:**

Search design complete, searches completed, additional articles obtained, duplicates removed, title and abstract review complete, risk of bias assessment complete. Data interpretation and manuscript preparation have begun.

**Named contact:** Dr. Michelle Ploughman.

**Named contact email:** michelle.ploughman@med.mun.ca.

**Named contact address:** Rehabilitation Research Unit of Newfoundland and Labrador, Rm 400, 100 Forest Rd, St. John's, NL, Canada, A1A 1E5.

**Named contact phone number:** (709) 777-2099.

**Organisational affiliation of the review:** Faculty of Medicine, Memorial University of Newfoundland.

**Review team members and their organisational affiliations:**

Dr. Michelle Ploughman, Rehabilitation Research Unit of Newfoundland and Labrador, Faculty of Medicine, Memorial University of Newfoundland, Canada (michelle.ploughman@med.mun.ca).
Mr. Nicholas Snow, Faculty of Medicine, Memorial University of Newfoundland, Canada (njsnow@mun.ca).

Dr. Katie P. Wadden, Faculty of Medicine, Memorial University of Newfoundland, Canada (kwadden@mun.ca).

Arthur R. de A. Chaves, Faculty of Medicine, Memorial University of Newfoundland, Canada

(ar.chaves@hotmail.com).

**Funding sources/sponsors:** N/A.

**Conflicts of interest:** N/A.

**Collaborators:** N/A.

**Review Methods**

**Review questions:**

What is the influence of multiple sclerosis (MS) on measures of corticospinal and intracortical excitability, as measured by transcranial magnetic stimulation (TMS), in combination with clinical measures of disease severity, as well as cognitive impairment, motor impairment, and/or fatigue, in human adults?

What are the relationships between the above outcome measures?

**Searches:**

We will search the Web of Science, MEDLINE, and Embase databases for published studies from 1985 (the first year of TMS publication [Barker, Jalinous, & Freeston, 1985]) onwards. Only articles published in English will be included. Reference lists of included articles will be searched to identify additional relevant studies. Relevant review articles will also be flagged, and reference lists examined, to identify further relevant studies. The searches will be re-run just prior to final data extraction to retrieve potential further studies for inclusion.

**Condition or domain being studied:**

Multiple sclerosis (MS).

**Participants/population:**

Studies including cross-sectional comparisons of adult human participants with MS and healthy control participants will be included. Comparisons at baseline by TMS in combination with clinical measures of disease severity, as well as cognitive impairment, motor impairment, and/or fatigue, will be included.

**Intervention(s), exposure(s):**

The present review will focus on observational data, or observations at baseline prior to intervention.

**Comparator(s)/control:**

Studies must include a healthy control group for comparison.

**Types of study to be included initially:**

Cross-sectional observational studies, as well as cohort studies and baseline (pre-intervention) data from controlled trials (randomized and non-) will be included.

**Primary outcome(s):**

TMS-based indices of motor system function (e.g., motor evoked potentials [MEPs], MEP recruitment curves, central motor conduction time [CMCT], cortical silent period [CSP], triple stimulation technique [TST]) and intracortical excitability (short- [SICI] and long-interval intracortical inhibition [LICI], short-interval intracortical facilitation [SICF], intracortical facilitation [ICF], ipsilateral silent period [iSP], interhemispheric inhibition [IHI]).

**Secondary outcome(s):**

Validated clinical assessments of disease severity, as well as cognitive impairment, motor impairment, and/or fatigue; relationships between TMS-based and clinical measures; moderators of TMS-clinical outcome relationships, including demographic factors (e.g., age, sex, disease status [primary- versus secondary-progressive versus relapsing-remitting]), lifestyle and medical factors (e.g., diet, exercise, rehabilitation, medication), and technical considerations (e.g., study design, TMS pulse parameters, clinical test or battery used).

**Data extraction (selection and coding):**

Only English-language full-text, peer-reviewed journal articles will be included. After removing duplicate records, titles and abstracts of search results will be evaluated for possible inclusion or exclusion by two reviewers (NJS, KPW). Relevant review papers will be flagged, and their reference lists examined for relevant records. Discrepancies will be resolved by reviewer consensus. The full-text of included articles will then be retrieved, and again evaluated for inclusion or exclusion, by two reviewers (NJS, KPW). Again, discrepancies will be resolved by consensus. After final inclusion of full-text articles, reference lists will be examined for further relevant articles. Data extraction and assessment of quality/bias will next be performed by two reviewers (NJS, KPW). Agreement will be reached by consensus, in the event of discrepancies across reviewers.

**Risk of bias (quality) assessment:**

The National Institutes of Health (NIH) Quality Assessment Tool for Observational Cohort and Cross-Sectional Studies ([link](https://www.nhlbi.nih.gov/health-pro/guidelines/in-develop/cardiovascular-risk-reduction/tools/cohort)) will be used to assess the quality of evidence. The existing version of the tool assesses risk of bias as high or low, based on responses to series of yes/no questions. To increase the stratification of risk to high, low, or unclear, the Cochrane Risk of Bias Tool ([link](http://handbook-5-1.cochrane.org/)) will be used to guide risk of bias judgements from the NIH tool. To assess the quality of TMS methods employed, a TMS checklist for assessing the methodological quality of studies will be used (Chipchase et al., 2012). Finally, to examine other important methodological or disease-specific confounding factors, background literature and included articles will be scanned to develop a list of additional criteria.

**Strategy for data synthesis:**

We will conduct a descriptive synthesis focused on the details of the observations, including disease status and participant demographics, as well as technical factors of TMS- and clinical-based outcomes. Inter-rater agreement among review stages will be assessed using percent differences and Cohen’s *κ* statistic (McHugh, 2012). Due to heterogeneity of the measures sought, as well as reporting methods across studies a meta-analysis will not be conducted.

**Analysis of subgroups or subsets:**

If the body of evidence is sufficiently large, descriptive subgroups will be established based on disease status or severity.

**General Information**

**Type of review and method of review:**

Diagnostic, prognostic.

**Health area of the review:**

Neurological.

**Language:**

English.

**Country:**

Canada.

**Other registration details:**

N/A.

**Reference and/or URL for published protocol:**

N/A.

**Dissemination plans:**

A manuscript will be submitted for publication to a relevant journal in the field.

**Keywords:**

Systematic review, transcranial magnetic stimulation, cognitive impairment, motor impairment, fatigue, multiple sclerosis.

**Details of any existing review of the same topic by the same authors:**

N/A.

**Current review status:**

Search design complete, searches completed, additional articles obtained, duplicates removed, title and abstract review complete, risk of bias assessment complete. Data interpretation and manuscript preparation have begun.

**Additional information:**

N/A.

**Details of final report/publication(s):**

N/A.

**PRISMA-P Checklist** [**(link)**](http://systematicreviewsjournal.biomedcentral.com/articles/10.1186/2046-4053-4-1#CR16)

**Rationale**

Multiple sclerosis (MS) is an autoimmune disease affecting the central nervous system (Kale, Agaoglu, Onder, & Tanik, 2009; Noseworthy, Lucchinetti, Rodriguez, & Weinshenker, 2000). Throughout the course of MS, demyelination of white matter in the brain and spinal cord occur (Lassmann, Brück, & Lucchinetti, 2007; Noseworthy et al., 2000), contributing to cognitive impairments (Hosseini, Flora, Banwell, & Till, 2014), motor impairments (Doble, Fisk, Fisher, Ritvo, & Murray, 1994), and fatigue (Conte et al., 2016). Results from existing systematic reviews indicate that across Canada, the prevalence of MS lies between approximately 50-300 cases per 100,000 individuals (Evans et al., 2013; Poppe, Wolfson, & Zhu, 2008), and ranks among the highest in the world (Evans et al., 2013; Kingwell et al., 2013; Poppe et al., 2008). Moreover, in Canada the prevalence of MS is higher in women, at a ratio of almost 3:1 (Evans et al., 2013); and nationwide, the incidence of MS appears to be increasing over time (Evans et al., 2013). Although there is inter-individual variability in disease progression, changes in myelination can be observed early in the disease course (Kale et al., 2009), and many individuals experience more permanent disability as the disease progresses (Lassmann et al., 2007; McDonald et al., 2001). Indeed, MS has been cited as the most common cause of nontraumatic disability acquired in young adults (Noseworthy et al., 2000; Pugliatti et al., 2006).

To date, numerous works have employed transcranial magnetic stimulation (TMS) to examine the integrity of the motor system in persons with MS, in relation to disease severity (Kale et al., 2009; Neva et al., 2016; Tataroglu, Genc, Idiman, Cakmur, & Idiman, 2003). Indeed, TMS has been used to study corticomotor characteristics of MS for over 30 years (Hess, Mills, & Murray, 1986). Several position stands and clinical guidelines have also been established to advise on the diagnostic and prognostic efficacy of TMS for individuals with MS (Chen et al., 2008; Groppa et al., 2012; Lefaucheur et al., 2014; Rossini et al., 2015). However, few works have combined assessments involving TMS with clinical measures of cognitive impairment (Cucurachi, Immovilli, Granella, Pavesi, & Cattaneo, 2008), motor impairment (Nantes et al., 2016; Zeller et al., 2011), and/or fatigue (Conte et al., 2016). Moreover, while limited work has summarized diagnostic and prognostic qualities of TMS in MS research (Brown, Neva, Ledwell, & Boyd, 2014; Rossini et al., 2015; Simpson & Macdonell, 2015), no existing review has comprehensively evaluated the literature, encompassing both neurophysiological and clinical characteristics of MS. To better understand the pathophysiology of MS in relation to behavioural outcomes, as well as to inform clinical decision-making, it is imperative to summarize the diagnostic and prognostic utility of TMS in relation to cognitive and motor assessments. Therefore, we aim to examine which TMS measures best characterize impairments in MS, as well as whether TMS can be considered a tool to measure impairments in sub-clinical MS.

**Objectives**

The primary objective of this review is to explore the impact of MS on motor system integrity (as indexed by TMS-based measures), in combination with disease severity, as well as cognitive impairment, motor impairment, and/or fatigue.

Our secondary objective is to summarize relationships between the above outcome measures.

**References**

Barker, A. T., Jalinous, R., & Freeston, I. L. (1985). Non-invasive magnetic stimulation of human motor cortex. *Lancet*, *1*(8437), 1106–1107.

Brown, K. E., Neva, J. L., Ledwell, N. M., & Boyd, L. (2014). Use of transcranial magnetic stimulation in the treatment of selected movement disorders. *Degenerative Neurological and Neuromuscular Disease*, *Volume 4*(December 2014), 133. https://doi.org/10.2147/DNND.S70079

Chen, R., Cros, D., Curra, A., Di Lazzaro, V., Lefaucheur, J. P., Magistris, M. R., … Ziemann, U. (2008). The clinical diagnostic utility of transcranial magnetic stimulation: Report of an IFCN committee. *Clinical Neurophysiology*, *119*(3), 504–532. https://doi.org/10.1016/j.clinph.2007.10.014

Chipchase, L., Schabrun, S., Cohen, L., Hodges, P., Ridding, M., Rothwell, J., … Ziemann, U. (2012). A checklist for assessing the methodological quality of studies using transcranial magnetic stimulation to study the motor system: An international consensus study. *Clinical Neurophysiology*, *123*(9), 1698–1704. https://doi.org/10.1016/j.clinph.2012.05.003

Conte, A., Li Voti, P., Pontecorvo, S., Quartuccio, M. E., Baione, V., Rocchi, L., … Berardelli, A. (2016). Attention-related changes in short-term cortical plasticity help to explain fatigue in multiple sclerosis. *Multiple Sclerosis Journal*, *22*(10), 1359–1366. https://doi.org/10.1177/1352458515619780

Cucurachi, L., Immovilli, P., Granella, F., Pavesi, G., & Cattaneo, L. (2008). Short-latency afferent inhibition predicts verbal memory performance in patients with multiple sclerosis. *Journal of Neurology*, *255*(12), 1949–1956. https://doi.org/10.1007/s00415-008-0041-5

Doble, S. E., Fisk, J. D., Fisher, A. G., Ritvo, P. G., & Murray, T. J. (1994). Functional competence of community-dwelling persons with multiple sclerosis using the assessment of motor and process skills. *Archives of Physical Medicine and Rehabilitation*, *75*(8), 843–851.

Evans, C., Beland, S. G., Kulaga, S., Wolfson, C., Kingwell, E., Marriott, J., … Marrie, R. A. (2013). Incidence and prevalence of multiple sclerosis in the Americas: A systematic review. *Neuroepidemiology*, *40*(3), 195–210. https://doi.org/10.1159/000342779

Groppa, S., Oliviero, A., Eisen, A., Quartarone, A., Cohen, L. G., Mall, V., … Siebner, H. R. (2012). A practical guide to diagnostic transcranial magnetic stimulation: Report of an IFCN committee. *Clinical Neurophysiology*, *123*(5), 858–882. https://doi.org/10.1016/j.clinph.2012.01.010

Hess, C. W., Mills, K. R., & Murray, N. M. F. (1986). Measurement of central motor conduction in multiple sclerosis by magnetic brain stimulation. *The Lancet*, (August), 355–358.

Hosseini, B., Flora, D. B., Banwell, B. L., & Till, C. (2014). Age of onset as a moderator of cognitive decline in pediatric-onset multiple sclerosis. *Journal of the International Neuropsychological Society*, *20*(8), 796–804. https://doi.org/10.1017/S1355617714000642

Kale, N., Agaoglu, J., Onder, G., & Tanik, O. (2009). Correlation between disability and transcranial magnetic stimulation abnormalities in patients with multiple sclerosis. *Journal of Clinical Neuroscience*, *16*(11), 1439–1442. https://doi.org/10.1016/j.jocn.2009.03.009

Kingwell, E., Marriott, J. J., Jetté, N., Pringsheim, T., Makhani, N., Morrow, S. A., … Marrie, R. A. (2013). Incidence and prevalence of multiple sclerosis in Europe: a systematic review. *BMC Neurology*, *13*(1), 128. https://doi.org/10.1186/1471-2377-13-128

Lassmann, H., Brück, W., & Lucchinetti, C. F. (2007). The immunopathology of multiple sclerosis: An overview. *Brain Pathology*, *17*(2), 210–218. https://doi.org/10.1111/j.1750-3639.2007.00064.x

Lefaucheur, J.-P., Andre-Obadia, N., Antal, A., Ayache, S. S., Baeken, C., Benninger, D. H., … Garcia-Larrea, L. (2014). Evidence-based guidelines on the therapeutic use of repetitive transcranial magnetic stimulation (rTMS). *Clinical Neurophysiology : Official Journal of the International Federation of Clinical Neurophysiology*, *125*(11), 2150–2206. https://doi.org/10.1016/j.clinph.2014.05.021

McDonald, W. I., Compston, A., Edan, G., Goodkin, D., Hartung, H.-P., Lublin, F. D., … Wolinsky, J. S. (2001). Recommended diagnostic criteria for multiple sclerosis: guidelines from the International Panel on the diagnosis of multiple sclerosis. *Annals of Neurology*, *50*, 121–127. https://doi.org/10.1002/ana.1032

McHugh, M. L. (2012). Interrater reliability: the kappa statistic. *Biochemia Medica*, *22*(3), 276–282. https://doi.org/10.11613/BM.2012.031

Nantes, J. C., Zhong, J., Holmes, S. A., Whatley, B., Narayanan, S., Lapierre, Y., … Koski, L. (2016). Intracortical inhibition abnormality during the remission phase of multiple sclerosis is related to upper limb dexterity and lesions. *Clinical Neurophysiology*, *127*, 1503–1511. https://doi.org/10.1016/j.clinph.2016.11.022

Neva, J. L., Lakhani, B., Brown, K. E., Wadden, K. P., Mang, C. S., Ledwell, N. M. H., … Boyd, L. A. (2016). Multiple measures of corticospinal excitability are associated with clinical features of multiple sclerosis. *Behavioural Brain Research*, *297*, 187–195. https://doi.org/10.1016/j.bbr.2015.10.015.

Noseworthy, J. H., Lucchinetti, C., Rodriguez, M., & Weinshenker, B. G. (2000). Multiple sclerosis. *New England Journal of Medicine*, *343*, 938–952. https://doi.org/10.1056/NEJM200009283431307

Poppe, A. Y., Wolfson, C., & Zhu, B. (2008). Prevalence of multiple sclerosis in Canada: a systematic review. *The Canadian Journal of Neurological Sciences. Le Journal Canadien Des Sciences Neurologiques*, *35*(5), 593–601. https://doi.org/10.1017/S0317167100009380

Pugliatti, M., Rosati, G., Carton, H., Riise, T., Drulovic, J., Vécsei, L., & Milanov, I. (2006). The epidemiology of multiple sclerosis in Europe. *European Journal of Neurology*, *13*(7), 700–722. https://doi.org/10.1111/j.1468-1331.2006.01342.x

Rossini, P. M., Burke, D., Chen, R., Cohen, L. G., Daskalakis, Z., Di Iorio, R., … Ziemann, U. (2015). Non-invasive electrical and magnetic stimulation of the brain, spinal cord, roots and peripheral nerves: Basic principles and procedures for routine clinical and research application. An updated report from an I.F.C.N. Committee. *Clinical Neurophysiology*, *126*(6), 1071–1107. https://doi.org/10.1016/j.clinph.2015.02.001

Simpson, M., & Macdonell, R. (2015). The use of transcranial magnetic stimulation in diagnosis, prognostication and treatment evaluation in multiple sclerosis. *Multiple Sclerosis and Related Disorders*, *4*(5), 430–436. https://doi.org/10.1016/j.msard.2015.06.014

Tataroglu, C., Genc, A., Idiman, E., Cakmur, R., & Idiman, F. (2003). Cortical silent period and motor evoked potentials in patients with multiple sclerosis. *Clinical Neurology and Neurosurgery*, *105*(2), 105–110.

Zeller, D., Dang, S.-Y., Stefan, K., Biller, A., Bartsch, A., Saur, D., … Classen, J. (2011). Functional role of ipsilateral motor areas in multiple sclerosis. *Journal of Neurology, Neurosurgery & Psychiatry*, *82*(5), 578–583. https://doi.org/10.1136/jnnp.2010.219964

**Appendix A – Search Protocol**

**General Overview**

1. Searches will be conducted in Web of Science, Embase and MEDLINE databases from 1985 onwards, and the results will be combined. We will initially eliminate duplicates and screen the titles and abstracts of the remaining records against inclusion and exclusion criteria. Any obvious papers will be removed. The number of records flagged for removal, as well as the reasons for exclusion, will be recorded.
2. During the above phase, we will also flag any relevant reviews and review reference lists for relevant articles. Relevant articles will be measured against inclusion and exclusion criteria, and recorded, as above.
3. After title and abstract review, we will include the full-text versions of any articles not eliminated. These records will again be measured against inclusion and exclusion criteria. The number of eliminated records and reasons for omission will again be recorded.
4. The remaining articles will be preserved for quality/risk of bias assessment, as well as data extraction. Reference lists of the included full-text articles will be scanned for relevant records. Relevant articles will be measured against inclusion and exclusion criteria, and recorded, as above.
5. Quality/risk of bias assessment will be performed in accordance with established systematic review protocol (NIH Quality Assessment Tool for Observational Cohort, Cross-Sectional Studies, Cochrane Risk of Bias Tool, and TMS checklist for assessing the methodological quality of studies).

**Inclusion/Exclusion Criteria**

**Inclusion Criteria:**

- Full-text peer-reviewed manuscripts, published in English, will be included.
- Studies must compare human participants with a primary diagnosis of multiple sclerosis with motor symptoms, and healthy control participants.
- Multiple sclerosis diagnosis must be definite, based on neurologist examination.
- Studies must utilize transcranial magnetic stimulation for measurement purposes (i.e., no plasticity-inducing protocols), in combination with validated clinical measures examining disease severity, as well as cognitive impairment, motor impairment, and/or fatigue.
- Clinical scales must be used outcome measures, and be reported as individual data, or using measures of central tendency (e.g., mean, median) and variation (e.g., standard deviation, range).
- Demographic data (i.e., age, sex) for both patient and control groups must be reported as individual data or using measures of central tendency (e.g., mean, median) and variation (e.g., standard deviation, range).
- Studies must include the Expanded Disability Status Scale as a measure of disease severity.
- TMS assessments will use surface electromyographic measurements from upper-limb muscles, in conjunction with TMS at a scalp site (supra-spinal level).
- Studies will be observational and cross-sectional, or can include baseline (i.e., pre-intervention) data from interventional or longitudinal studies, provided baseline statistical comparisons are made between MS and control groups.
- Cross-sectional (observational) studies, as well as baseline (pre-intervention) data from cohort studies and controlled trials (randomized and non-) will be included.

**Exclusion Criteria:**

- Papers not published in English will be excluded.
- Conference proceedings, abstracts, and case studies will not be included.
- Animal studies will be excluded.
- Studies examining samples of pediatric (< 18 years of age) participants will not be included.
- TMS studies of non-upper- (e.g., urinary bladder, anal sphincter), or studies of lower-limb muscles will be excluded.

**Search Logic Overview**

**Search Conditions:**

1. Multiple sclerosis,
2. “Transcranial magnetic stimulation” OR “TMS” OR “magnetic stimulation”,
3. **Select studies that meet condition 1 AND condition 2,**
4. **Manually search for studies examining disease severity, cognitive impairment, motor impairment, and/or fatigue to increase the number of search hits.**
   - **Including the search terms AND (“cognit*” OR “motor*” OR “sever*”) severely limited the number of resultant articles at initial search.**

**Search:**

'multiple sclerosis' AND ('transcranial magnetic stimulation' OR 'tms' OR 'magnetic stimulation')

**Appendix B – Search Result Processing**

**Initial Search Date:** September 8, 2017

**Initial Search Results**

Web of Science: n = 708

Embase: n = 491

MEDLINE: n = 267

**Total: N = 1466**

**Import**

Searches exported as .txt/.csv files.

Files imported into Microsoft Excel.

Columns rearranged to produce: Article Number (used to track articles in spread sheet), Authors, Title, Abstract, Journal, Year, Volume, Issue, Start Page, End Page, e-page (for online-based journals that do not use page ranges [e.g., BMC, PLoS One]), DOI (digital object identifier).

Columns added: Include (Yes / No / Uncertain [to be reviewed later and followed up with principal investigator] / Review [used to flag relevant review papers]), Comments (reasons for, e.g., exclusion or uncertainty).

**Title and Abstract Review of Initial Search**

Remove any duplicate items.

**Total: N = 958**

Add any items from other sources (e.g., reviews).

**Total from Reviews: N = 41**

**Total from Full-texts: N = 23**

**Total from Manual Search: N = 8**

**Total: N = 72**

Total records.

**Total: N = 1030**

Review titles and abstracts against inclusion and exclusion criteria.

Reviewer 1

**Y = 132**

**N = 878**

**R = 11**

**U = 0**

Reviewer 2

**Y = 119**

**N = 834**

**R = 0**

**U = 67**

Consensus

**Y = 151**

**N = 857**

**R = 12**

**U = 0**

**Full-text Review of Initial Search**

Reviewer 1

**Y = 29**

**N = 94**

**R = 1**

**U = 27**

Reviewer 2

**Y = 23**

**N = 98**

**R = 1**

**U = 29**

Consensus

**Y = 29**

**N = 121**

**R = 1**

**U = 0**

**Additional Records of Initial Search**

Scan reference lists of all full-text articles marked Y, under the Include column.

Scan reference lists of review articles marked R, under the Include column.

Review titles/abstracts and full-texts of any included non-redundant records, based on above reference lists.

**Total Y: N = 0**

**Total N: N = 23**

**Articles Included for Risk of Bias Assessment and Data Extraction**

**Total Y: N = 25**

**Total N: N = 54**

**Secondary Search Date** November 29, 2018

**Secondary Search Results (New Records)**

Web of Science: n = 77

Embase: n = 26

MEDLINE: n = 34

**Total: N = 137**

**Title and Abstract Review of Secondary Search**

Remove any duplicate items.

**Total: N = 40**

Add any items from other sources (e.g., reviews).

**Total from Reviews: N = 1**

**Total from Full-texts: N = 1**

**Total from Manual Search: N = 1**

**Total: N = 3**

Total records.

**Total: N = 100**

Review titles and abstracts against inclusion and exclusion criteria.

Reviewer 1

**Y = 9**

**N = 76**

**R = 11**

**U = 4**

Reviewer 2

**Y = 18**

**N = 72**

**R = 4**

**U = 6**

Consensus

**Y = 11**

**N = 83**

**R = 6**

**U = 0**

**Full-text Review of Secondary Search**

Reviewer 1

**Y = 3**

**N = 7**

**R = 0**

**U = 0**

Reviewer 2

**Y = 6**

**N = 4**

**R = 0**

**U = 0**

Consensus

**Y = 2**

**N = 8**

**R = 0**

**U = 0**

**Additional Records of Secondary Search**

Scan reference lists of all full-text articles marked Y, under the Include column.

Scan reference lists of review articles marked R, under the Include column.

Review titles/abstracts and full-texts of any included non-redundant records, based on above reference lists.

**Total Y: N = 1**

**Total N: N = 2**

**Secondary Search Articles Included for Risk of Bias Assessment and Data Extraction**

**Total Y: N = 2**

**Total N: N = 98**

**Appendix C – PRISMA 2009 Flow Diagram**

Studies included in qualitative synthesis
(n = 30)

Full-text articles assessed for eligibility
(n = 162)

Records screened
(n = 1130)

Records after duplicates removed
(n = 1130)

## Identification

## Eligibility

## Included

## Screening

Full-text articles excluded, with reasons
(n = 132)

- Conference (n = 40),
- No clinical measures (n = 12),
- No healthy control group (n = 10),
- No TMS (n = 3),
- No patient demographics (n = 2),
- No control demographics (n = 1),
- No surface EMG electrodes (n = 3),
- No EDSS (n = 12),
- Review paper (n = 4),
- Baseline TMS not compared (n = 10),
- Healthy controls only (n = 1),
- Cannot obtain article (n = 13),
- Disorders other than MS (n = 9),
- Upper limb not examined (n = 6),
- Not published in English (n = 1),
- Secondary data analysis (n = 3),
- MS criteria not defined (n = 1),
- Commentary (n = 1)

Records excluded
(n = 968)

Additional records identified through other sources
(reference lists, review papers)
(n = 75)

Records identified through database searching
(n = 1603)
