# Supplementary material for: Transcranial Magnetic Stimulation as a Potential Biomarker in Multiple Sclerosis: A Systematic Review with Recommendations for Future Research
Source: Neural Plast. 2019 Sep 16;2019:6430596. doi: 10.1155/2019/6430596 (PMC6766108; doi:10.1155/2019/6430596)
Supplement: Supplementary Materials — Figure S1: key potential confounding variable findings. In-depth descriptions of the TMS and clinical outcomes employed in reviewed studies can be found in Tables S1 and S2, respectively. See Tables S3 and S4 for results pertaining to article screening and data extraction, respectively. Original review protocol can be found in Supplemental Methods 1-2. [file 6430596.f1.zip › Supplemental Methods 2 - Review Protocol.pdf]

National Institute for Health Research

University of York
Centre for Reviews and Dissemination

Systematic review

1. * Review title.

Give the working title of the review, for example the one used for obtaining funding. Ideally the title should state succinctly the interventions or exposures being reviewed and the associated health or social problems. Where appropriate, the title should use the PI(E)COS structure to contain information on the Participants, Intervention (or Exposure) and Comparison groups, the Outcomes to be measured and Study designs to be included.

Using transcranial magnetic stimulation to detect cognitive, motor, and fatigue outcomes in multiple sclerosis

2. Original language title.

For reviews in languages other than English, this field should be used to enter the title in the language of the review. This will be displayed together with the English language title.

3. * Anticipated or actual start date.

Give the date when the systematic review commenced, or is expected to commence.

15/09/2017

4. * Anticipated completion date.

Give the date by which the review is expected to be completed.

28/02/2019

5. * Stage of review at time of this submission.

Indicate the stage of progress of the review by ticking the relevant Started and Completed boxes. Additional information may be added in the free text box provided.
Please note: Reviews that have progressed beyond the point of completing data extraction at the time of initial registration are not eligible for inclusion in PROSPERO. Should evidence of incorrect status and/or completion date being supplied at the time of submission come to light, the content of the PROSPERO record will be removed leaving only the title and named contact details and a statement that inaccuracies in the stage of the review date had been identified.
This field should be updated when any amendments are made to a published record and on completion and publication of the review. If this field was pre-populated from the initial screening questions then you are not able to edit it until the record is published.

The review has not yet started: No

Review stage	Started	Completed
Preliminary searches	Yes	Yes
Piloting of the study selection process	Yes	Yes
Formal screening of search results against eligibility criteria	Yes	Yes
Data extraction	Yes	Yes
Risk of bias (quality) assessment	Yes	Yes
Data analysis	Yes	Yes

Provide any other relevant information about the stage of the review here (e.g. Funded proposal, protocol not yet finalised).

Search design complete, searches completed, additional articles obtained, duplicates removed, title and abstract review complete, risk of bias assessment complete. Data interpretation and manuscript preparation been completed. The draft manuscript has been submitted for publication.

Search design complete, searches completed, additional articles obtained, duplicates removed, title and abstract review complete, risk of bias assessment complete. Data interpretation and manuscript preparation been completed. The draft manuscript has been submitted for publication.

6. * Named contact.

The named contact acts as the guarantor for the accuracy of the information presented in the register record.

Dr. Michelle Ploughman

Email salutation (e.g. "Dr Smith" or "Joanne") for correspondence:

Dr. Ploughman

7. * Named contact email.

Give the electronic mail address of the named contact.

michelle.ploughman@med.mun.ca

8. Named contact address

Give the full postal address for the named contact.

Rehabilitation Research Unit of Newfoundland and Labrador, Rm 400, 100 Forest Rd, St. John's, NL, Canada, A1A 1E5

9. Named contact phone number.

Give the telephone number for the named contact, including international dialling code.

(709) 777-2099

10. * Organisational affiliation of the review.

Full title of the organisational affiliations for this review and website address if available. This field may be

completed as 'None' if the review is not affiliated to any organisation.

Memorial University of Newfoundland

Organisation web address:

http://www.med.mun.ca/RRUNL/Home.aspx

11. * Review team members and their organisational affiliations.

Give the title, first name, last name and the organisational affiliations of each member of the review team. Affiliation refers to groups or organisations to which review team members belong.

Mr Nicholas Snow. Memorial University of Newfoundland
Dr Katie Wadden. Memorial University of Newfoundland
Mr Arthur Chaves. Memorial University of Newfoundland
Dr Michelle Ploughman. Memorial University of Newfoundland

12. * Funding sources/sponsors.

Give details of the individuals, organizations, groups or other legal entities who take responsibility for initiating, managing, sponsoring and/or financing the review. Include any unique identification numbers assigned to the review by the individuals or bodies listed.

None.

13. * Conflicts of interest.

List any conditions that could lead to actual or perceived undue influence on judgements concerning the main topic investigated in the review.

None

14. Collaborators.

Give the name and affiliation of any individuals or organisations who are working on the review but who are not listed as review team members.

15. * Review question.

State the question(s) to be addressed by the review, clearly and precisely. Review questions may be specific or broad. It may be appropriate to break very broad questions down into a series of related more specific questions. Questions may be framed or refined using PI(E)COS where relevant.

What is the influence of multiple sclerosis (MS) on measures of corticospinal and intracortical excitability, as measured by transcranial magnetic stimulation (TMS), in combination with clinical measures of disease severity, as well as cognitive impairment, motor impairment, and/or fatigue, in human adults?

What are the relationships between the above outcome measures?

16. * Searches.

Give details of the sources to be searched, search dates (from and to), and any restrictions (e.g. language or publication period). The full search strategy is not required, but may be supplied as a link or attachment.

Search Overview:

Searches were conducted in Web of Science, Embase and MEDLINE databases from 1985 (the first year of TMS publication [Barker, Jalinous, and Freeston, 1985]) onwards. Only articles published in English were be

included. Results were combined and duplicates removed.

Search Criteria:

1. Multiple sclerosis,
2. “Transcranial magnetic stimulation” OR “TMS” OR “magnetic stimulation”,

3. Select studies that meet condition 1 AND condition 2,

4. Manually search for studies examining disease severity, cognitive impairment, motor impairment, and/or fatigue to increase the number of search hits.

Final Search:

'multiple sclerosis' AND ('transcranial magnetic stimulation' OR 'tms' OR 'magnetic stimulation')

17. URL to search strategy.

Give a link to a published pdf/word document detailing either the search strategy or an example of a search strategy for a specific database if available (including the keywords that will be used in the search strategies), or upload your search strategy.Do NOT provide links to your search results.

Alternatively, upload your search strategy to CRD in pdf format. Please note that by doing so you are consenting to the file being made publicly accessible.

Do not make this file publicly available until the review is complete

18. * Condition or domain being studied.

Give a short description of the disease, condition or healthcare domain being studied. This could include health and wellbeing outcomes.

Multiple sclerosis (MS).

19. * Participants/population.

Give summary criteria for the participants or populations being studied by the review. The preferred format includes details of both inclusion and exclusion criteria.

Studies including cross-sectional comparisons of adult human participants with MS and healthy control participants will be included. Comparisons at baseline by TMS in combination with clinical measures of disease severity, as well as cognitive impairment, motor impairment, and/or fatigue, will be included.

20. * Intervention(s), exposure(s).

Give full and clear descriptions or definitions of the nature of the interventions or the exposures to be reviewed.

The present review will focus on observational data, or observations at baseline prior to intervention.

21. * Comparator(s)/control.

Where relevant, give details of the alternatives against which the main subject/topic of the review will be compared (e.g. another intervention or a non-exposed control group). The preferred format includes details of both inclusion and exclusion criteria.

Studies must include a healthy control group for comparison.

22. * Types of study to be included.

Give details of the types of study (study designs) eligible for inclusion in the review. If there are no restrictions on the types of study design eligible for inclusion, or certain study types are excluded, this should be stated. The preferred format includes details of both inclusion and exclusion criteria.

Cross-sectional observational studies, as well as cohort studies and baseline (pre-intervention) data from controlled trials (randomized and non-) will be included.

23. Context.

Give summary details of the setting and other relevant characteristics which help define the inclusion or exclusion criteria.

Multiple sclerosis (MS) is a disease affecting the central nervous system (Noseworthy, Lucchinetti, Rodriguez, and Weinshenker, 2000). Throughout the course of MS, demyelination of white matter in the brain and spinal cord (Lassmann, Brück, and Lucchinetti, 2007; Noseworthy et al., 2000) contribute to cognitive impairments (Hosseini, Flora, Banwell, and Till, 2014), motor impairments (Doble, Fisk, Fisher, Ritvo, and Murray, 1994), and fatigue (Conte et al., 2016). Although there is inter-individual variability in disease progression, changes in myelination can be observed early in the disease course (Kale et al., 2009), and many individuals experience permanent disability as the disease progresses (Lassmann et al., 2007; McDonald et al., 2001).

To date, numerous works have employed transcranial magnetic stimulation (TMS) to examine the integrity of the motor system in persons with MS (Kale et al., 2009; Neva et al., 2016; Tataroglu, Genc, Idiman, Cakmur, and Idiman, 2003). However, little work has have combined TMS assessments with clinical measures of disease severity and associated impairments (Conte et al., 2016; Cucurachi, Immovilli, Granella, Pavesi, and Cattaneo, 2008; Nantes et al., 2016; Zeller et al., 2011). Moreover, limited work has summarized diagnostic and prognostic qualities of TMS in MS research (Boyd, Brown, Ledwell, and Neva, 2014; Rossini et al., 2015; Simpson and Macdonell, 2015), and no existing review has comprehensively evaluated the literature, encompassing both neurophysiological and clinical characteristics of MS. To better understand the pathophysiology of MS in relation to behavioural outcomes, we aim to examine which TMS measures best characterize impairments in MS.

24. * Main outcome(s).

Give the pre-specified main (most important) outcomes of the review, including details of how the outcome is defined and measured and when these measurement are made, if these are part of the review inclusion criteria.

The primary objective of this review is to explore the impact of MS on motor system integrity (as indexed by TMS-based measures), in combination with disease severity, as well as cognitive impairment, motor impairment, and/or fatigue.

Timing and effect measures

Cross-sectional comparisons between participants with MS and healthy control participants will be summarized as found in the source manuscripts.

25. * Additional outcome(s).

List the pre-specified additional outcomes of the review, with a similar level of detail to that required for main outcomes. Where there are no additional outcomes please state 'None' or 'Not applicable' as appropriate to the review

Our secondary objective is to summarize relationships between the above outcome measures.

Timing and effect measures

Simple correlations between TMS and clinical outcomes will be summarized as found in the source manuscripts.

26. * Data extraction (selection and coding).

Give the procedure for selecting studies for the review and extracting data, including the number of researchers involved and how discrepancies will be resolved. List the data to be extracted.

Only English-language full-text, peer-reviewed journal articles will be included. After removing duplicate records, titles and abstracts of search results will be evaluated for possible inclusion or exclusion by two reviewers (NJS, KPW). Relevant review papers will be flagged, and their reference lists examined for relevant records. Discrepancies will be resolved by reviewer consensus. The full-text of included articles will then be retrieved, and again evaluated for inclusion or exclusion, by two reviewers (NJS, KPW). Again, discrepancies will be resolved by consensus. After final inclusion of full-text articles, reference lists will be examined for further relevant articles. Data extraction and assessment of quality/bias will next be performed by two reviewers (NJS, KPW). Agreement will be reached by consensus, in the event of discrepancies across reviewers.

27. * Risk of bias (quality) assessment.

State whether and how risk of bias will be assessed (including the number of researchers involved and how discrepancies will be resolved), how the quality of individual studies will be assessed, and whether and how this will influence the planned synthesis.

The National Institutes of Health (NIH) Quality Assessment Tool for Observational Cohort and Cross-Sectional Studies will be used to assess the quality of evidence. The existing version of the tool assesses risk of bias as high or low, based on responses to series of yes/no questions. To increase the stratification of risk to high, low, or unclear, the Cochrane Risk of Bias Tool will be used to guide risk of bias judgements from the NIH tool. To assess the quality of TMS methods employed, a TMS checklist for assessing the methodological quality of studies will be used (Chipchase et al., 2012). Finally, to examine other important methodological or disease-specific confounding factors, background literature and included articles will be scanned to develop a list of additional criteria.

28. * Strategy for data synthesis.

Give the planned general approach to synthesis, e.g. whether aggregate or individual participant data will be used and whether a quantitative or narrative (descriptive) synthesis is planned. It is acceptable to state that a quantitative synthesis will be used if the included studies are sufficiently homogenous.

We will conduct a descriptive synthesis focused on the details of the observations, including disease status and participant demographics, as well as technical factors of TMS- and clinical-based outcomes. Inter-rater agreement among review stages will be assessed using percent differences and Cohen's ? statistic (McHugh, 2012). Due to heterogeneity of the measures sought, as well as reporting methods across studies a meta-analysis will not be conducted.

29. * Analysis of subgroups or subsets.

Give details of any plans for the separate presentation, exploration or analysis of different types of participants (e.g. by age, disease status, ethnicity, socioeconomic status, presence or absence or co-morbidities); different types of intervention (e.g. drug dose, presence or absence of particular components of intervention); different settings (e.g. country, acute or primary care sector, professional or family care); or different types of study (e.g. randomised or non-randomised).

If the body of evidence is sufficiently large, descriptive subgroups will be established based on disease status or severity.

30. * Type and method of review.

Select the type of review and the review method from the lists below. Select the health area(s) of interest for your review.

Type of review

Cost effectiveness
No

Diagnostic
Yes

Epidemiologic
No

Individual patient data (IPD) meta-analysis
No

Intervention
No

Meta-analysis
No

Methodology
No

Narrative synthesis
No

Network meta-analysis
No

Pre-clinical
No

Prevention
No

Prognostic
Yes

Prospective meta-analysis (PMA)
No

Review of reviews
No

Service delivery
No

Synthesis of qualitative studies
No

Systematic review
Yes

Other
No

Health area of the review

Alcohol/substance misuse/abuse
No

Blood and immune system
No

Cancer
No

Cardiovascular
No

Care of the elderly
No

Child health
No

Complementary therapies
No

Crime and justice
No

Dental
No

Digestive system
No

Ear, nose and throat
No

Education
No

Endocrine and metabolic disorders
No

Eye disorders
No

General interest
No

Genetics
No

Health inequalities/health equity
No

Infections and infestations
No

International development
No

Mental health and behavioural conditions
No

Musculoskeletal
No

Neurological
Yes

Nursing
No

Obstetrics and gynaecology
No

Oral health
No

Palliative care
No

Perioperative care
No

Physiotherapy
No

Pregnancy and childbirth
No

Public health (including social determinants of health)
No

Rehabilitation
No

Respiratory disorders
No

Service delivery
No

Skin disorders
No

Social care
No

Surgery
No

Tropical Medicine
No

Urological
No

Wounds, injuries and accidents
No

Violence and abuse
No

31. Language.

Select each language individually to add it to the list below, use the bin icon to remove any added in error.
English

There is not an English language summary

32. Country.

Select the country in which the review is being carried out from the drop down list. For multi-national collaborations select all the countries involved.

Canada

33. Other registration details.

Give the name of any organisation where the systematic review title or protocol is registered (such as with The Campbell Collaboration, or The Joanna Briggs Institute) together with any unique identification number assigned. (N.B. Registration details for Cochrane protocols will be automatically entered). If extracted data will be stored and made available through a repository such as the Systematic Review Data Repository (SRDR), details and a link should be included here. If none, leave blank.

34. Reference and/or URL for published protocol.

Give the citation and link for the published protocol, if there is one

Give the link to the published protocol.

Alternatively, upload your published protocol to CRD in pdf format. Please note that by doing so you are consenting to the file being made publicly accessible.

No I do not make this file publicly available until the review is complete

Please note that the information required in the PROSPERO registration form must be completed in full even if access to a protocol is given.

35. Dissemination plans.

Give brief details of plans for communicating essential messages from the review to the appropriate audiences.

A manuscript will be submitted for publication to a relevant journal in the field.

Do you intend to publish the review on completion?

Yes

36. Keywords.

Give words or phrases that best describe the review. Separate keywords with a semicolon or new line. Keywords will help users find the review in the Register (the words do not appear in the public record but are included in searches). Be as specific and precise as possible. Avoid acronyms and abbreviations unless these are in wide use.

Systematic review; Transcranial magnetic stimulation; Cognitive impairment; Motor impairment; Fatigue;

Multiple sclerosis

37. Details of any existing review of the same topic by the same authors.

Give details of earlier versions of the systematic review if an update of an existing review is being registered, including full bibliographic reference if possible.

38. * Current review status.

Review status should be updated when the review is completed and when it is published. For newregistrations the review must be Ongoing.

Please provide anticipated publication date

Review_Completed_not_published

39. Any additional information.

Provide any other information the review team feel is relevant to the registration of the review.

40. Details of final report/publication(s).

This field should be left empty until details of the completed review are available.

This review has been submitted for publication.

Give the link to the published review.
