# Supplementary material for: Transcranial Magnetic Stimulation as a Potential Biomarker in Multiple Sclerosis: A Systematic Review with Recommendations for Future Research
Source: Neural Plast. 2019 Sep 16;2019:6430596. doi: 10.1155/2019/6430596 (PMC6766108; doi:10.1155/2019/6430596)
Supplement: Supplementary Materials — Figure S1: key potential confounding variable findings. In-depth descriptions of the TMS and clinical outcomes employed in reviewed studies can be found in Tables S1 and S2, respectively. See Tables S3 and S4 for results pertaining to article screening and data extraction, respectively. Original review protocol can be found in Supplemental Methods 1-2. [file 6430596.f1.zip › Supplemental Table S1 - TMS measures.docx]

**Table S1.** Transcranial magnetic stimulation (TMS) measures employed in studies.

| **TMS Protocol** | **Stimulation Characteristics** | **Neural Mechanisms** | **Studies Utilizing** |
| --- | --- | --- | --- |
| **Single-pulse TMS** | | | |
| **Active Motor Threshold (AMT)** | Stimulator intensity necessary to elicit a MEP with a peak-to-peak amplitude of 100-200 μV in at least five of 10 consecutive trials, during a tonic contraction of the target muscle (10-20% MVC) [37,44,114,115]. Reported as % MSO. | Characterizes the most excitable elements of the corticospinal tract [33]. Reflects activity of VGSCs and AMPARs [98]. Influenced by Glu [98]. | Llufriu et al. [54],  Morgante et al. [70],  Neva et al. [74]. |
| **Cortical Silent Period (CSP)** | Quiescence in rectified EMG trace after a MEP [37], when TMS is delivered during a sustained contraction of the target muscle contralateral to M1 (20% MVC) [74,116–118]. Level of contraction and size of MEP do not impact CSP, but CSP duration tends to increase linearly with TMS stimulus intensity [44,119]. Reported as duration or latency. | Generated by both spinal (recurrent inhibition, refractoriness of spinal motor neurons, post-synaptic inhibition) and intracortical (GABA_B_Rs) inhibitory mechanisms [35,44,98,108,109]. | Nantes et al. [42],  Nantes et al. [71],  Neva et al. [74],  Santarnecchi et al. [76],  Tataroglu et al. [85],  Vucic et al. [87],  White et al. [78]. |
| **Ipsilateral Silent Period (iSP)** | Disruption of background EMG activity, following the TMS pulse, during a tonic contraction (10-50% MVC) of the muscle ipsilateral to the target motor area stimulated [65,74,118,120–123]. Reported as duration, latency, amplitude, or conduction time. | Represents inhibition across cerebral hemispheres [124] via transcallosal [123] or other commissural pathways [125]. Generated in part by GABA_B_Rs, albeit underlying mechanisms not fully understood [33]. | Lenzi et al. [73],  Llufriu et al. [54],  Neva et al. [74],  Schmierer et al. [65]. |
| **Motor Evoked Potential (MEP)** | Deflection in EMG trace of a target muscle following the delivery of a TMS pulse (120% RMT or SI_1 mV_) to the M1 target area [44]. Measured in an active or resting muscle. MEP amplitude tends to increase in a sigmoidal relationship with TMS stimulus intensity [126,127]. Reported as amplitude, latency, or duration; or number of turns or abnormalities. Other studies examine the MEP recruitment curve, which records MEP amplitude at a variety of stimulus intensities. | Reflects summation of action potentials in corticospinal axons which synapse with spinal motor neurons [37,107]. Amplitude reflects corticospinal excitability; latency is indicative of corticomotor latency; duration highlights synchronization of motor neuron action potentials; number of turns, which highlights polyphasic activation of multiple neuron pools; or abnormalities [37,44,68,128,129]. The slope of recruitment curve represents the gain properties of the motor system, representing the excitability of multiple populations of motor neurons in a corticospinal representation [126,127]. Regulated by Glu, GABA, 5-HT, and NE [44]. | Bridoux et al. [66],  Conte et al. [8],  Cucurachi et al. [43],  Facchetti et al. [68],  Firmin et al. [88],  Ho et al. [72],  Ingram et al. [82],  Kale et al. [69],  Lenzi et al. [73],  Liepert et al. [81],  Morgante et al. [70],  Nantes et al. [42],  Nantes et al. [71],  Neva et al. [74],  Perretti et al. [75],  Tataroglu et al. [85],  Tataroglu et al. [86],  Vucic et al. [87],  White et al. [78],  Zeller et al. [63],  Zeller et al. [79]. |
| **Resting Motor Threshold (RMT)** | Lowest TMS stimulus intensity capable of evoking a MEP in a resting target muscle [114]. Stimulator intensity at which a MEP with a peak-to-peak amplitude of 50 μV is obtained in at least five of 10 consecutive trials, in the resting muscle [44,114]. Reported as % MSO. | Characterizes “bias” level of the corticospinal pathway [115,127] and represents the activity of the most excitable “core” of corticospinal and motor neurons [33,127]. Reflects activity of VGSCs and AMPA receptors [98].  Influenced by Glu [98]. | Cucurachi et al. [43],  Ho et al. [72],  Hulst et al. [80],  Lenzi et al. [73],  Liepert et al. [81],  Llufriu et al. [54],  Morgante et al. [70],  Nantes et al. [42],  Nantes et al. [71],  Neva et al. [74],  Perretti et al. [75],  Schmierer et al. [65],  Vucic et al. [87],  Zeller et al. [79],  Zipser et al. [64]. |
| **Paired-pulse TMS** | | | |
| **Central Motor Conduction Time (CMCT)** | Determined by subtracting spinal cord- or brainstem-to-muscle latency from M1-to-muscle latency [35,37]. M1-to-muscle latency is considered the onset latency of the MEP in response of TMS performed over M1 [44]. Spinal cord-/brainstem-to-muscle latency is estimated in two ways [35,37,44]: 1) stimulating the spinal cord over the cervical region either electrically or with TMS [130,131]; or 2) electrically stimulating the efferent nerve innervating the target muscle, with the cathode proximal, to produce a brief and supramaximal F-wave [114,132,133]. Performed during relaxed conditions or throughout a slight isometric contraction of the target muscle [35,37]. Reported as difference between M1-to-muscle and spinal cord-/brainstem-to-muscle latencies. | CMCT is a measure of cortical output latency [33], used to estimate the conduction time of the corticospinal tract between M1 and brainstem or spinal motor neurons [35,37]. CMCT has been posited as one of the more clinically useful TMS methods in examinations of MS [35,37,93]. | Cucurachi et al. [43],  Facchetti et al. [68],  Firmin et al. [88],  Ho et al. [72],  Ingram et al. [82],  Kale et al. [69],  Lenzi et al. [73],  Llufriu et al. [54],  Morgante et al. [70],  Sandroni et al. [83],  Schmierer et al. [65],  Tataroglu et al. [86],  Vucic et al. [87],  Wahl et al. [77],  White et al. [78],  Zeller et al. [79]. |
| **Dorsal Premotor Cortex-Primary Motor Cortex Interactions (PMd-M1)** | Dual-coil method in which CS is applied over PMd and a suprathreshold (SI_1 mV_) TS over M1 [67,134]. Depending on the intensity of the CS, either inhibition or facilitation of the conditioned MEP can be induced in the target muscle contralateral to M1 (ipsilateral to PMd) [67,134,135]. A suprathreshold CS will produce MEP inhibition, whereas a subthreshold CS will facilitate the MEP elicited by the TS [135]. Reported as ratio/% of unconditioned TS. | Effects driven by integration of the CS and TS in PMd and transmitted via transcallosal pathways [67,135]. Intracortical circuits mediating PMD-M1 interactions are distinct from M1 intracortical interneurons [135]. | Codecà et al. [67]. |
| **Interhemispheric Inhibition (IHI)** | Using two TMS coils, interhemispheric interactions between homologous M1 representations are examined in a relaxed muscle. A suprathreshold CS is delivered over one hemisphere and a suprathreshold TS (SI_1 mV_) is delivered over the other hemisphere [122]. The size and duration of the inhibitory effects of the CS are impacted by its intensity, such that the TS MEP can be inhibited or facilitated [122,134]; thus, a range of CS intensities are tested [77,122]. Interhemispheric facilitation is elicited at shorter ISIs (4-12 ms) while interhemispheric inhibition occurs at relatively longer ISIs (20-50 ms) [35,44,77,122,136,137]. Reported as ratio/% of unconditioned TS. | IHI is thought to be a cortical event [122,134,136,137], mediated by GABA_B_Rs [138]; although SIHI and LIHI seem to involve distinct intracortical circuits [136]. | Wahl et al. [77]. |
| **Intracortical Facilitation (ICF)** | Involves the delivery of a subthreshold CS, followed approximately 10-15 ms later by a suprathreshold TS while the participant is at rest [33,35,44,139]. Reported as ratio/% of unconditioned TS. | Recognized as a true measure of cortical facilitation [140]. Influenced by Glu intracortical networks [98,140]. Unclear whether ICF is an exclusively cortically-driven effect or if there are spinal contributions as well [35,107]. | Codecà et al. [67],  Liepert et al. [81],  Morgante et al. [70],  Nantes et al. [42],  Neva et al. [74],  Vucic et al. [87]. |
| **Long-interval Intracortical Inhibition (LICI)** | Utilizes two suprathreshold stimuli (120% RMT or SI_1 mV_), separated by an ISI of 50-200 ms while the participant is relaxed [44,141–143]. Reported as ratio/% of unconditioned TS. | LICI is considered a primarily cortical phenomenon [144], mediated by GABA_B_Rs receptors [98,145]. | Codecà et al. [67],  Nantes et al. [42]. |
| **Short-interval Intracortical Facilitation (SICF)** | Tested using a subthreshold or suprathreshold CS, paired with a TS that is either subthreshold or suprathreshold [67,146–149]. SICF measurement requires the use of several discrete ISIs including 1.1-1.5 ms, 2.3-3.0 ms, and 4.1-4.5 ms [146,148], which have been used in varying combinations throughout the literature [44,67,121]. Reported as ratio/% of unconditioned TS. | The ISIs employed in SICF research are intended to coincide with discrete I-waves produced by the trans-synaptic activation of corticospinal neurons following the TMS pulse [37,107,147]. SICF is thought to be a cortical facilitatory phenomenon [35,44,146], primarily controlled by GABA_A_Rs [98,107]. | Codecà et al. [67],  Ho et al. [72]. |
| **Short-interval Intracortical Inhibition (SICI)** | Measured when a sub-threshold CS is followed 1-5 ms later by a supra-threshold TS while the participant is at rest [37,114,139]. Magnitude of SICI depends on the CS and TS intensity [143,150]. Reported as ratio/% of unconditioned TS. | SICI is thought to be a cortical phenomenon [144]. Stimuli delivered using a 1 ms ISI presumably assess synaptic inhibition modulated by extra-synaptic levels of GABA [151] or by neuronal refractoriness [152]. ISIs ≥ 2.5 ms examine GABA_A_R-mediated inhibition [98,107]. | Codecà et al. [67],  Liepert et al. [81],  Morgante et al. [70],  Nantes et al. [42],  Nantes et al. [71],  Neva et al. [74],  Vucic et al. [87]. |
| **Short-latency Afferent Inhibition (SAI)** | Produced by delivering an electrical CS over a peripheral afferent nerve (median nerve) contralateral to M1, approximately 20-25 ms prior to delivering a suprathreshold TMS TS over the target M1 area in a relaxed participant [44,153–155]. The result is a reduction in the size of the subsequent MEP [155]. Reported as ratio/% of unconditioned TS. | SAI is said to be a cortical measure involving thalamocortical projections [153,155], mediated by muscarinic Ach receptors and GABA_A_Rs [98]. SAI is considered a measure of sensorimotor integration [33]. | Cucurachi et al. [43]. |
| **Triple Stimulation Technique (TST)** | Entails the delivery of a suprathreshold TMS pulse over the M1 representation of the target muscle, supramaximal electrical stimulation over the distal part of the peripheral nerve supplying the target muscle, and a second supramaximal electrical stimulation over the proximal part of the same nerve (Erb’s point) [156,157]. ISIs are individualized for each participant to ensure action potentials induced by TMS collide with the corticospinal volleys resulting from peripheral nerve stimulations [156,157]. A TST test curve, obtained as per above, is compared to a control curve derived from triple stimulation of the peripheral neve [35]. Reported as amplitude ratio, latency, or latency variability. | This method results in “re-synchronization” of corticospinal action potentials at the level of the peripheral motor neuron [37] and overcomes trial-to-trial variability in MEPs that is caused by phase cancellation and asynchronous firing of corticospinal motor neurons [37]. The main utility of TST is to examine corticospinal conduction [157,158], and neurological disorders affecting central conduction tend to produce smaller TST amplitude ratios and different latency characteristics than HCs [88,157]. | Firmin et al. [88],  Scheidegger et al. [84]. |

MVC, maximum voluntary isometric contraction; MSO, maximal stimulator output; VGSC, voltage-gated sodium channel; AMPAR, α-amino-3-hydroxy-5-methyl-4-isoxazolepropionic acid receptor (glutamate receptor); Glu, glutamate; EMG, electromyogram; MEP, motor evoked potential; M1, primary motor cortex; GABA_B_R, γ-aminobutyric acid receptor B; SI_1 mV_, stimulator intensity required to elicit a MEP with ~1 mV peak-to-peak amplitude; GABA, γ-aminobutyric acid; 5-HT, serotonin; NE, norepinephrine; CS, conditioning stimulus; TS, test stimulus; ISI, interstimulus interval; GABA_A_R, γ-aminobutyric acid receptor A; Ach, acetylcholine; HC, healthy control.

**Table 4.** Clinical measures employed in studies.

| **Clinical Measure** | **Description** | **Characteristic Tested** | **Studies Utilizing** |
| --- | --- | --- | --- |
| **Disease Severity** | | | |
| **Expanded Disability Status Scale (EDSS)** | Administered by a trained examiner and used with observations concerning gait and use of assistive devices [51]. The EDSS is rated from 0.0-10.0, in increments of 0.5, where 0.0 indicates no disability and 10.0 denotes that the individual has died due to MS [51]. Scores from 1.0-4.5 describe persons who can to walk without any mobility aid [51]. Scores from 5.0-9.5 refer to impairments in walking, ranging from being able to walk 200 m without aid (5.0), to being confined to bed and unable to communicate or swallow (9.5) [51]. | The EDSS is an ordinal scale based on the standard neurological examination, and is used characterize clinical level of disability in MS [51]. Disease severity is sometimes stratified by EDSS score, such that, 0.0-1.0 indicates no disability, 2.0-3.0 refers to mild to moderate disability, and ≥ 4.0 means severe disability [55,67]. | Bridoux et al. [66],  Codecà et al. [67],  Conte et al. [8],  Cucurachi et al. [43],  Facchetti et al. [68],  Firmin et al. [88],  Ho et al. [72],  Hulst et al. [80],  Ingram et al. [82],  Kale et al. [69],  Lenzi et al. [73],  Liepert et al. [81],  Llufriu et al. [54],  Morgante et al. [70],  Nantes et al. [42],  Nantes et al. [71],  Neva et al. [74],  Perretti et al. [75],  Sandroni et al. [83],  Santarnecchi et al. [76],  Scheidegger et al. [84],  Schmierer et al. [65],  Tataroglu et al. [85],  Tataroglu et al. [86],  Vucic et al. [87],  Wahl et al. [77],  White et al. [78],  Zeller et al. [63],  Zeller et al. [79],  Zipser et al. [64]. |
| **Kurtzke’s Functional Systems Scores (Kurtzke FSS)** | Administered by a trained rater [159]. The Functional Systems Scores are a set of eight subscales, each rated from 0-9 in discrete increments of 1, where greater score denotes more severe disability [159]. The subscales include: pyramidal function, cerebellar function, brainstem function, sensory function, bowel and bladder function, visual function, cerebral/mental function, and other features noted by the examiner [159]. Scores can be reported separately or as a composite [159]. | The Kurtzke FSS is an ordinal scale based on the standard neurological examination, and is used characterize clinical level of disability in MS [159]. | Facchetti et al. [68],  Tataroglu et al. [85],  Tataroglu et al. [86]. |
| **Multiple Sclerosis Functional Composite (MSFC)** | Administered by a trained examiner [160]. This battery contains three primary measures: Timed 25-foot Walk (T25FW), Nine-hole Peg Test (9HPT), and Paced Auditory Serial Addition Test-3 seconds (PASAT3). Each test item can be scored separately, or the results from all three tests can be yield a composite score [160]. | The MSFC is a standardized, quantitative, multidimensional instrument intended to reflect clinical severity of MS [160]. Sub-scales assess leg function/ambulation (T25FW), arm/hand function (9HPT), and cognitive function (PASAT3) [160]. | Conte et al. [8],  Llufriu et al. [54],  Nantes et al. [42],  Nantes et al. [71]. |
| **Cognitive Impairment** | | | |
| **Brief Repeatable Battery (BRB)** | Administered by a trained examiner [161] and includes elements of the selective reminding (SRT) (verbal memory), spatial recall test (SPART) (visual memory), symbol digit modalities test (SDMT) (attention, visual precision search, processing speed, executive functions), paced auditory serial addition test (PASAT) (maintenance of attention, processing speed, working memory), world list generation (WLG) (associative verbal fluency), and Stroop test (ST) (selective attention) [54,161]. Subscales can be scored independently, collapsed across specific cognitive domains (i.e., verbal memory, visual memory, etc.), or combined for a total composite [54,161]. Scores can also be converted to *z*-scores [54]. | The BRB is a standardized collection of scales intended to measure cognitive impairment in persons with MS [161]. | Llufriu et al. [54]. |
| **California Verbal Learning Test (VLGT)** | Given by a trained administrator [162]. A list of 16 nouns is read aloud, with a 1-second ISI [162]. The list is read over five trials, after each of which the participant attempts to recall as many nouns as possible [162]. Participants are also provided with an interference list of words with similar meaning [162]. Both recall and recognition of the original list are tested at different intervals [162]. A learning curve with learning parameters, response errors, and interference effects is used for scoring [162]. | The LVGT is a widely used test of episodic verbal learning and memory [163]. This test has moderate test-retest reliability in persons with MS [164]. | Hulst et al. [80]. |
| **Digit Span** | Measured by a trained rater [165]. Sequences of digits are presented in forward and reverse order, and the participant recalls the sequences [165–167]. Two trials are presented at each sequence length, beginning with two digits, until either the participant fails to recall either trial or the maximal span length is reached (nine forward, eight backward) [165–167]. The total number of lists recalled correctly is combined across forward span and backward spans to give total correct score [165–167]. | Digit Span is a commonly used test of short-term verbal memory [165–167]. This test has been evaluated in MS as part of larger tests of cognition [168]. | Hulst et al. [80]. |
| **Frontal Assessment Battery (FAB)** | Delivered by a trained rater [169]. The FAB utilizes six subscales that examine conceptualization (similarities test), mental flexibility (verbal fluency test), motor programming (Luria motor sequences), sensitivity to interference (conflicting instructions), inhibitory control (go-no go test), and environmental autonomy (prehension behaviour) [169]. Each of the subscales is rated from 0-3, and the sum of the scores is interpreted; 18 is the maximum (best) score and < 12 indicates cognitive impairment [169]. | The FAB is a valid and reliable test designed to be sensitive to cognitive impairment due to frontal lobe dysfunction [169]. | Conte et al. [8]. |
| **Letter Digit Substitution Test (LDST)** | Assessed by a trained rater [170]. The test is administered in a visual or auditory format [171]. In this test digits 1 to 9 are associated with a corresponding letter [170]. After practicing the task, participants must replace randomized letters with the appropriate digit as quickly as possible [170]. Test scoring is based on the number of correct letter-digit substitutions made in 60 seconds [170]. | The LDST provides a measure of information processing speed, as well as visual or auditory memory [170,171]. | Hulst et al. [80]. |
| **Location Learning Test (LLT)** | Administered by a trained rater [172,173]. Participants are shown an array of images five times each, for 30 seconds at a time [172,173]. After each presentation, and 15 minutes after the last presentation, participants must relocate the images to their correct position on an empty grid [172,173]. For every trial a Displacement Score is measured consisting of the sum of the errors made for each object placement on that trial [172,173]. A Total Displacement Score combines the Displacement Scores on the first five learning trials [172,173]. A Learning Index represents the relative difference in performance between trials [172,173]. A Delayed Recall Score considers the difference between trial last and the 15-minute delayed trial [172,173]. | The LLT is a test of visuospatial learning and memory [172]. | Hulst et al. [80]. |
| **Letter Number Sequencing (LNS)** | Provided by a trained rater in either auditory or visual form [165,166,174,175]. The participant is presented a series of letters and digits in a non-systematic order. Following the presentation, the participant must report back the stimuli, with the letters in alphabetical order and the digits in ascending order. Scoring is based on correctness of responses. | The LNS is a test of auditory or visual working memory and attention [165,166,174,175]. | Hulst et al. [80]. |
| **Mini Mental State Exam (MMSE)** | Thirty-point questionnaire examining aspects of cognitive function including registration, attention, calculation, recall, language, ability to follow simple commands, and orientation [176,177]. Scoring is relative to age- and education-based norms [176]. | The MMSE is used to quantify cognitive impairment [176,177]. | Sandroni et al. [83]. |
| **N-Back** | Computer-based task [178,179]. Participants press one of two buttons, denoting target and non-target, in response to a target (letter) that matches a stimulus presented zero, one, two, or three stimuli previously. Scoring is based on reaction time and correctness of responses in each condition. | The N-back test is a processing speed and working memory task [178,179]. | Hulst et al. [80]. |
| **Paced Auditory Serial Addition (PASAT-2 / PASAT-3)** | Administered by a trained rater [180]. A series of digits is presented, either visually or aurally, and the two most recent digits must be summed [180]. An ISI of 2 (PASAT-2) or 3 seconds (PASAT-3) separates each digit [180]. Scoring is based on the number of correct responses for each trial or the total number of correct responses over all trials [180]. The PASAT is part of the MSFC [160] and BRB [161]. | The PASAT is a test of processing speed and working memory [180]. | Conte et al. [8],  Cucurachi et al. [43],  Llufriu et al. [54],  Nantes et al. [71]. |
| **Posner Test** | Computer-based task involving responding to visual stimuli presented in one of two possible locations on the computer screen [181]. Prior to the stimulus, a visual cue directs the participant’s attention either to the correct location (valid cue) or an incorrect location (invalid cue). There are a proportionate number of valid and invalid cues, and non-cued stimuli, which are randomly interspersed [8,181]. Performance is based on correct responses and reaction time, and can be compared across cue conditions [8,181]. | The Posner Test is an index of attention [8,181]. | Conte et al. [8]. |
| **Selective Reminding Test- (SRT-LTS / SRT-CLTR / SRT-D)** | Administered by a trained rater [182]. The participant hears a list of 12 unrelated words and must recall as many words as possible. Every subsequent trial involves the administrator reminding the participant only of those words the participant did not recall on the previous trial. Trials of recall and selective reminding continue until the participant can correctly recall all 12 words on three consecutive trials, or until 12 trials have been completed. Scores are provided for words recalled from long-term storage (SRT-LTS), consistently from long-term retrieval (SRT-CLTR), and delayed recall (SRT-D). The SRT is part of the BRB [161]. | The SRT examines verbal memory and learning [182], and can distinguish memory retrieval from long-term storage versus and short-term recall [182]. | Conte et al. [8],  Cucurachi et al. [43],  Llufriu et al. [54]. |
| **Spatial Recall Test (SPART / SPART-D)** | Administered by a trained rater [161]. In this test, 6 × 6 checkerboard displaying a pattern of 10 checkers is placed in front of the participant for 10 seconds. The participant tries to reproduce the pattern using a blank checkerboard and 10 checkers. This occurs for three trials, plus a 15-minute delayed-recall trial. Scoring is based on the number of correctly-placed checkers over the first three trials (SPART), as well as during the delayed-recall trial (SPART-D). The SPART is part of the BRB [161]. | The SPART [161] assesses visuospatial learning in MS research [183]. | Conte et al. [8],  Cucurachi et al. [43],  Llufriu et al. [54]. |
| **Stroop Test** | Given by a trained experimenter [184]. Participants are instructed to read aloud a list of colour names as quickly as possible, leaving no errors uncorrected. The task utilizes five words (red, blue, green, brown, purple) and their matching ink colours. Each ink colour appears twice in each row and column on 10-word × 10-word card. The task examines the effect of incompatible ink colour on reading words aloud and measures response time. The Stroop Test is a component of the BRB [161]. | The Stroop Test is a measure of selective attention [54,161]. | Conte et al. [8]. |
| **Symbol Digit Modalities Test (SDMT)** | Provided by a trained administer [185]. The participant is given 90 seconds to pair specific numbers with given geometric figures, based on a reference key provided by the experimenter. Participants can give either written or oral responses. Studies in MS tend to use spoken responses over written [186]. The administrator uses a predetermined scoring form. The SDMT is a component of the BRB [161]. | The SDMT provides an index of attention, visual precision search, processing speed, and executive functions [161,185]. This is a valid and reliable tool in MS research, with particular sensitivity to slowed information processing [186]. | Conte et al. [8],  Cucurachi et al. [43],  Llufriu et al. [54]. |
| **Word List Generation (WLG)** | Administered by a trained examined [187]. Participants are asked to say as many different words as possible that begin with a specific letter (letter fluency); they are allotted 60 seconds for each trial [187–189]. Participants cannot say proper nouns nor variations of the same word root [187–189]. Next, participants must say as many words as possible from a specific category (category fluency); 60 seconds are allowed for each trial [187–189]. This test is part of the BRB [161]. | The WLG task assesses verbal fluency, including category fluency (ability to list objects in different categories) and letter fluency (ability to list different words beginning with the same letter) [187–189]. This task has also been suggested to measure semantic memory and retrieval from long-term memory storage [189]. | Hulst et al. [80]. |
| **Motor Impairment** | | | |
| **Grip Strength** | A handgrip dynamometer is used to measure Grip in kg, using a standard protocol and under the supervision of a trained rater. Results can be compared to norms, between individuals, or across limbs. Grip strength can also be measured as pinch grip strength or as a maximum voluntary isometric contraction (MVC). | Grip strength can be used to describe hand function, and to index overall body strength, in persons with MS [190]. | Liepert et al. [81],  Nantes et al. [71],  Perretti et al. [75]. |
| **Medical Research Council (MRC) Strength Scale** | Rated by a trained administrator [191]. The experimenter grades muscle strength on a scale of 0-5, relative to the maximum expected strength. A score of 0 indicates no contraction of the muscle, while 5 indicates normal strength. This test is performed separately for muscles of interest [82]. The MRC scale is part of a standard neurological examination [192]. | The MRC Scale is an ordinal scale used to examine muscle strength [191]. | Ingram et al. [82],  Kale et al. [69]. |
| **Modified Ashworth Scale (MAS)** | Conducted by a trained examiner [193]. This ordinal scale uses discrete ratings of 1 and is scored from 0-4; 0 reflects normal tone and 4 indicates that the tested muscle is rigid during flexion or extension. While the participant is in a supine position, the examiner passively flexes and extends joints of interest, providing a rating for each. Spasticity testing is a component of a standard neurological examination [192]. | The MAS is used to assess muscle spasticity or resistance to passive movement [192,193]. | Ho et al. [72]. |
| **Nine-hole Peg Test (9HPT)** | Administered by a trained examiner [160]. The participant sits at a table with a small, shallow container holding nine pegs and a block containing nine empty holes. On a start command, the participant picks up and places each of the nine pegs in the nine holes as fast as possible, one at a time. The participant then removes them as quickly as possible, placing them into their container. The total time to complete the task is recorded. Two consecutive trials with the dominant hand are immediately followed by two consecutive trials with the non-dominant hand. Both trials for each hand are averaged and reported separately. The 9HPT is part of the MSFC [160]. | The 9HPT is used to examine finger dexterity [160]. | Nantes et al. [42],  Nantes et al. [71],  Wahl et al. [77],  Zeller et al. [63]. |
| **Reflexes** | Examined by a trained rater [82,194]. A tendon is tapped briskly by a reflex hammer and the resultant muscle contraction is given an ordinal score of 0-4, where 0 reflexes abnormal hyporeflexia and 4 denotes abnormal hyperreflexia. A score of 2 is normal. Deep tendon reflexes are part of a standard neurological examination [164, 166]. | Deep tendon reflexes can be used to assess the presence or severity of upper- versus lower-motor neuron lesions [192]. | Ingram et al. [82],  Kale et al. [69]. |
| **Timed 25-foot Walk (T25FW)** | Administered by a trained examiner [195,196]. The participant is instructed to walk as fast and safely as possible across a marked 25-foot linear course, using an assistive device if necessary [195]. The participant is timed walking the course twice and the two trials are averaged [195]. Scoring can be expressed as time or speed [195], or as part of the Ambulatory Index [196], a 10-point scale that assesses mobility based on time and degree of assistance required during the T25FW. The T25FW is a component of the MSFC [160]. | The T25FW is related to walking performance and lower extremity function [195]. This test has strong test-retest reliability in MS across a wide range of disability levels and is sensitive to intervention effects in longitudinal studies [195]. | Nantes et al. [71]. |
| **Fatigue** | | | |
| **Fatigue Impact Scale (FIS)** | Self-report measure used to examine participants’ perceptions of how fatigue impacts their quality of life [197]. The scale is comprised of 40 items that are scored from 0 (no problem) to 4 (extreme problem), providing a total composite score of 0-160. The FIS contains subdomains that reflect perceived impact on cognitive (concentration, memory, thinking, organization of thoughts), physical (motivation, effort, stamina, coordination), and psychosocial functioning (isolation, emotions, workload, coping) (10 items/40 points each). | The FIS is a subjective measure of fatigue [197]. | White et al. [78]. |
| **Fatigue Severity Scale (FSS)** | Self-report measure that uses a series of 7-point scales to examine the severity and impact of subjective feelings of fatigue [198]. In response to each of the nine statements provided in the FSS, a rating of 1 indicates strong disagreement while 7 refers to strong agreement. A total score < 36 indicates that the individual may not be suffering fatigue, whereas > 36 suggests that one may be experiencing fatigue and should seek medical counsel [198]. | The FSS provides a subjective measure of fatigue [198]. | Bridoux et al. [66],  Perretti et al. [75]. |
| **Modified Fatigue Impairment Scale (MFIS)** | Abbreviated version of the FIS that has been adapted for persons with MS [197,199]. As for the FIS, the MFIS contains cognitive (9 items/36 points), physical (10 items/40 points), and psychosocial (2 items/8 points) subscales; however, this test only contains 21 items, and can be rated out of a total 0-84 points [197,199]. | The MFIS is a subjective measure of fatigue [197,199]. | Conte et al. [8],  Vucic et al. [87]. |

ISI, interstimulus interval.
