# Supplementary material for: Transcranial Magnetic Stimulation as a Potential Biomarker in Multiple Sclerosis: A Systematic Review with Recommendations for Future Research
Source: Neural Plast. 2019 Sep 16;2019:6430596. doi: 10.1155/2019/6430596 (PMC6766108; doi:10.1155/2019/6430596)
Supplement: Supplementary Materials — Figure S1: key potential confounding variable findings. In-depth descriptions of the TMS and clinical outcomes employed in reviewed studies can be found in Tables S1 and S2, respectively. See Tables S3 and S4 for results pertaining to article screening and data extraction, respectively. Original review protocol can be found in Supplemental Methods 1-2. [file 6430596.f1.zip › Supplemental Table S2 - Clinical measures.docx]

**Table S2.** Clinical measures employed in studies.

| **Clinical Measure** | **Description** | **Characteristic Tested** | **Studies Utilizing** |
| --- | --- | --- | --- |
| **Disease Severity** | | | |
| **Expanded Disability Status Scale (EDSS)** | Administered by a trained examiner and used with observations concerning gait and use of assistive devices [51]. The EDSS is rated from 0.0-10.0, in increments of 0.5, where 0.0 indicates no disability and 10.0 denotes that the individual has died due to MS [51]. Scores from 1.0-4.5 describe persons who can to walk without any mobility aid [51]. Scores from 5.0-9.5 refer to impairments in walking, ranging from being able to walk 200 m without aid (5.0), to being confined to bed and unable to communicate or swallow (9.5) [51]. | The EDSS is an ordinal scale based on the standard neurological examination, and is used characterize clinical level of disability in MS [51]. Disease severity is sometimes stratified by EDSS score, such that, 0.0-1.0 indicates no disability, 2.0-3.0 refers to mild to moderate disability, and ≥ 4.0 means severe disability [55,67]. | Bridoux et al. [66],  Codecà et al. [67],  Conte et al. [8],  Cucurachi et al. [43],  Facchetti et al. [68],  Firmin et al. [88],  Ho et al. [72],  Hulst et al. [80],  Ingram et al. [82],  Kale et al. [69],  Lenzi et al. [73],  Liepert et al. [81],  Llufriu et al. [54],  Morgante et al. [70],  Nantes et al. [42],  Nantes et al. [71],  Neva et al. [74],  Perretti et al. [75],  Sandroni et al. [83],  Santarnecchi et al. [76],  Scheidegger et al. [84],  Schmierer et al. [65],  Tataroglu et al. [85],  Tataroglu et al. [86],  Vucic et al. [87],  Wahl et al. [77],  White et al. [78],  Zeller et al. [63],  Zeller et al. [79],  Zipser et al. [64]. |
| **Kurtzke’s Functional Systems Scores (Kurtzke FSS)** | Administered by a trained rater [159]. The Functional Systems Scores are a set of eight subscales, each rated from 0-9 in discrete increments of 1, where greater score denotes more severe disability [159]. The subscales include: pyramidal function, cerebellar function, brainstem function, sensory function, bowel and bladder function, visual function, cerebral/mental function, and other features noted by the examiner [159]. Scores can be reported separately or as a composite [159]. | The Kurtzke FSS is an ordinal scale based on the standard neurological examination, and is used characterize clinical level of disability in MS [159]. | Facchetti et al. [68],  Tataroglu et al. [85],  Tataroglu et al. [86]. |
| **Multiple Sclerosis Functional Composite (MSFC)** | Administered by a trained examiner [160]. This battery contains three primary measures: Timed 25-foot Walk (T25FW), Nine-hole Peg Test (9HPT), and Paced Auditory Serial Addition Test-3 seconds (PASAT3). Each test item can be scored separately, or the results from all three tests can be yield a composite score [160]. | The MSFC is a standardized, quantitative, multidimensional instrument intended to reflect clinical severity of MS [160]. Sub-scales assess leg function/ambulation (T25FW), arm/hand function (9HPT), and cognitive function (PASAT3) [160]. | Conte et al. [8],  Llufriu et al. [54],  Nantes et al. [42],  Nantes et al. [71]. |
| **Cognitive Impairment** | | | |
| **Brief Repeatable Battery (BRB)** | Administered by a trained examiner [161] and includes elements of the selective reminding (SRT) (verbal memory), spatial recall test (SPART) (visual memory), symbol digit modalities test (SDMT) (attention, visual precision search, processing speed, executive functions), paced auditory serial addition test (PASAT) (maintenance of attention, processing speed, working memory), world list generation (WLG) (associative verbal fluency), and Stroop test (ST) (selective attention) [54,161]. Subscales can be scored independently, collapsed across specific cognitive domains (i.e., verbal memory, visual memory, etc.), or combined for a total composite [54,161]. Scores can also be converted to *z*-scores [54]. | The BRB is a standardized collection of scales intended to measure cognitive impairment in persons with MS [161]. | Llufriu et al. [54]. |
| **California Verbal Learning Test (VLGT)** | Given by a trained administrator [162]. A list of 16 nouns is read aloud, with a 1-second ISI [162]. The list is read over five trials, after each of which the participant attempts to recall as many nouns as possible [162]. Participants are also provided with an interference list of words with similar meaning [162]. Both recall and recognition of the original list are tested at different intervals [162]. A learning curve with learning parameters, response errors, and interference effects is used for scoring [162]. | The LVGT is a widely used test of episodic verbal learning and memory [163]. This test has moderate test-retest reliability in persons with MS [164]. | Hulst et al. [80]. |
| **Digit Span** | Measured by a trained rater [165]. Sequences of digits are presented in forward and reverse order, and the participant recalls the sequences [165–167]. Two trials are presented at each sequence length, beginning with two digits, until either the participant fails to recall either trial or the maximal span length is reached (nine forward, eight backward) [165–167]. The total number of lists recalled correctly is combined across forward span and backward spans to give total correct score [165–167]. | Digit Span is a commonly used test of short-term verbal memory [165–167]. This test has been evaluated in MS as part of larger tests of cognition [168]. | Hulst et al. [80]. |
| **Frontal Assessment Battery (FAB)** | Delivered by a trained rater [169]. The FAB utilizes six subscales that examine conceptualization (similarities test), mental flexibility (verbal fluency test), motor programming (Luria motor sequences), sensitivity to interference (conflicting instructions), inhibitory control (go-no go test), and environmental autonomy (prehension behaviour) [169]. Each of the subscales is rated from 0-3, and the sum of the scores is interpreted; 18 is the maximum (best) score and < 12 indicates cognitive impairment [169]. | The FAB is a valid and reliable test designed to be sensitive to cognitive impairment due to frontal lobe dysfunction [169]. | Conte et al. [8]. |
| **Letter Digit Substitution Test (LDST)** | Assessed by a trained rater [170]. The test is administered in a visual or auditory format [171]. In this test digits 1 to 9 are associated with a corresponding letter [170]. After practicing the task, participants must replace randomized letters with the appropriate digit as quickly as possible [170]. Test scoring is based on the number of correct letter-digit substitutions made in 60 seconds [170]. | The LDST provides a measure of information processing speed, as well as visual or auditory memory [170,171]. | Hulst et al. [80]. |
| **Location Learning Test (LLT)** | Administered by a trained rater [172,173]. Participants are shown an array of images five times each, for 30 seconds at a time [172,173]. After each presentation, and 15 minutes after the last presentation, participants must relocate the images to their correct position on an empty grid [172,173]. For every trial a Displacement Score is measured consisting of the sum of the errors made for each object placement on that trial [172,173]. A Total Displacement Score combines the Displacement Scores on the first five learning trials [172,173]. A Learning Index represents the relative difference in performance between trials [172,173]. A Delayed Recall Score considers the difference between trial last and the 15-minute delayed trial [172,173]. | The LLT is a test of visuospatial learning and memory [172]. | Hulst et al. [80]. |
| **Letter Number Sequencing (LNS)** | Provided by a trained rater in either auditory or visual form [165,166,174,175]. The participant is presented a series of letters and digits in a non-systematic order. Following the presentation, the participant must report back the stimuli, with the letters in alphabetical order and the digits in ascending order. Scoring is based on correctness of responses. | The LNS is a test of auditory or visual working memory and attention [165,166,174,175]. | Hulst et al. [80]. |
| **Mini Mental State Exam (MMSE)** | Thirty-point questionnaire examining aspects of cognitive function including registration, attention, calculation, recall, language, ability to follow simple commands, and orientation [176,177]. Scoring is relative to age- and education-based norms [176]. | The MMSE is used to quantify cognitive impairment [176,177]. | Sandroni et al. [83]. |
| **N-Back** | Computer-based task [178,179]. Participants press one of two buttons, denoting target and non-target, in response to a target (letter) that matches a stimulus presented zero, one, two, or three stimuli previously. Scoring is based on reaction time and correctness of responses in each condition. | The N-back test is a processing speed and working memory task [178,179]. | Hulst et al. [80]. |
| **Paced Auditory Serial Addition (PASAT-2 / PASAT-3)** | Administered by a trained rater [180]. A series of digits is presented, either visually or aurally, and the two most recent digits must be summed [180]. An ISI of 2 (PASAT-2) or 3 seconds (PASAT-3) separates each digit [180]. Scoring is based on the number of correct responses for each trial or the total number of correct responses over all trials [180]. The PASAT is part of the MSFC [160] and BRB [161]. | The PASAT is a test of processing speed and working memory [180]. | Conte et al. [8],  Cucurachi et al. [43],  Llufriu et al. [54],  Nantes et al. [71]. |
| **Posner Test** | Computer-based task involving responding to visual stimuli presented in one of two possible locations on the computer screen [181]. Prior to the stimulus, a visual cue directs the participant’s attention either to the correct location (valid cue) or an incorrect location (invalid cue). There are a proportionate number of valid and invalid cues, and non-cued stimuli, which are randomly interspersed [8,181]. Performance is based on correct responses and reaction time, and can be compared across cue conditions [8,181]. | The Posner Test is an index of attention [8,181]. | Conte et al. [8]. |
| **Selective Reminding Test- (SRT-LTS / SRT-CLTR / SRT-D)** | Administered by a trained rater [182]. The participant hears a list of 12 unrelated words and must recall as many words as possible. Every subsequent trial involves the administrator reminding the participant only of those words the participant did not recall on the previous trial. Trials of recall and selective reminding continue until the participant can correctly recall all 12 words on three consecutive trials, or until 12 trials have been completed. Scores are provided for words recalled from long-term storage (SRT-LTS), consistently from long-term retrieval (SRT-CLTR), and delayed recall (SRT-D). The SRT is part of the BRB [161]. | The SRT examines verbal memory and learning [182], and can distinguish memory retrieval from long-term storage versus and short-term recall [182]. | Conte et al. [8],  Cucurachi et al. [43],  Llufriu et al. [54]. |
| **Spatial Recall Test (SPART / SPART-D)** | Administered by a trained rater [161]. In this test, 6 × 6 checkerboard displaying a pattern of 10 checkers is placed in front of the participant for 10 seconds. The participant tries to reproduce the pattern using a blank checkerboard and 10 checkers. This occurs for three trials, plus a 15-minute delayed-recall trial. Scoring is based on the number of correctly-placed checkers over the first three trials (SPART), as well as during the delayed-recall trial (SPART-D). The SPART is part of the BRB [161]. | The SPART [161] assesses visuospatial learning in MS research [183]. | Conte et al. [8],  Cucurachi et al. [43],  Llufriu et al. [54]. |
| **Stroop Test** | Given by a trained experimenter [184]. Participants are instructed to read aloud a list of colour names as quickly as possible, leaving no errors uncorrected. The task utilizes five words (red, blue, green, brown, purple) and their matching ink colours. Each ink colour appears twice in each row and column on 10-word × 10-word card. The task examines the effect of incompatible ink colour on reading words aloud and measures response time. The Stroop Test is a component of the BRB [161]. | The Stroop Test is a measure of selective attention [54,161]. | Conte et al. [8]. |
| **Symbol Digit Modalities Test (SDMT)** | Provided by a trained administer [185]. The participant is given 90 seconds to pair specific numbers with given geometric figures, based on a reference key provided by the experimenter. Participants can give either written or oral responses. Studies in MS tend to use spoken responses over written [186]. The administrator uses a predetermined scoring form. The SDMT is a component of the BRB [161]. | The SDMT provides an index of attention, visual precision search, processing speed, and executive functions [161,185]. This is a valid and reliable tool in MS research, with particular sensitivity to slowed information processing [186]. | Conte et al. [8],  Cucurachi et al. [43],  Llufriu et al. [54]. |
| **Word List Generation (WLG)** | Administered by a trained examined [187]. Participants are asked to say as many different words as possible that begin with a specific letter (letter fluency); they are allotted 60 seconds for each trial [187–189]. Participants cannot say proper nouns nor variations of the same word root [187–189]. Next, participants must say as many words as possible from a specific category (category fluency); 60 seconds are allowed for each trial [187–189]. This test is part of the BRB [161]. | The WLG task assesses verbal fluency, including category fluency (ability to list objects in different categories) and letter fluency (ability to list different words beginning with the same letter) [187–189]. This task has also been suggested to measure semantic memory and retrieval from long-term memory storage [189]. | Hulst et al. [80]. |
| **Motor Impairment** | | | |
| **Grip Strength** | A handgrip dynamometer is used to measure Grip in kg, using a standard protocol and under the supervision of a trained rater. Results can be compared to norms, between individuals, or across limbs. Grip strength can also be measured as pinch grip strength or as a maximum voluntary isometric contraction (MVC). | Grip strength can be used to describe hand function, and to index overall body strength, in persons with MS [190]. | Liepert et al. [81],  Nantes et al. [71],  Perretti et al. [75]. |
| **Medical Research Council (MRC) Strength Scale** | Rated by a trained administrator [191]. The experimenter grades muscle strength on a scale of 0-5, relative to the maximum expected strength. A score of 0 indicates no contraction of the muscle, while 5 indicates normal strength. This test is performed separately for muscles of interest [82]. The MRC scale is part of a standard neurological examination [192]. | The MRC Scale is an ordinal scale used to examine muscle strength [191]. | Ingram et al. [82],  Kale et al. [69]. |
| **Modified Ashworth Scale (MAS)** | Conducted by a trained examiner [193]. This ordinal scale uses discrete ratings of 1 and is scored from 0-4; 0 reflects normal tone and 4 indicates that the tested muscle is rigid during flexion or extension. While the participant is in a supine position, the examiner passively flexes and extends joints of interest, providing a rating for each. Spasticity testing is a component of a standard neurological examination [192]. | The MAS is used to assess muscle spasticity or resistance to passive movement [192,193]. | Ho et al. [72]. |
| **Nine-hole Peg Test (9HPT)** | Administered by a trained examiner [160]. The participant sits at a table with a small, shallow container holding nine pegs and a block containing nine empty holes. On a start command, the participant picks up and places each of the nine pegs in the nine holes as fast as possible, one at a time. The participant then removes them as quickly as possible, placing them into their container. The total time to complete the task is recorded. Two consecutive trials with the dominant hand are immediately followed by two consecutive trials with the non-dominant hand. Both trials for each hand are averaged and reported separately. The 9HPT is part of the MSFC [160]. | The 9HPT is used to examine finger dexterity [160]. | Nantes et al. [42],  Nantes et al. [71],  Wahl et al. [77],  Zeller et al. [63]. |
| **Reflexes** | Examined by a trained rater [82,194]. A tendon is tapped briskly by a reflex hammer and the resultant muscle contraction is given an ordinal score of 0-4, where 0 reflexes abnormal hyporeflexia and 4 denotes abnormal hyperreflexia. A score of 2 is normal. Deep tendon reflexes are part of a standard neurological examination [164, 166]. | Deep tendon reflexes can be used to assess the presence or severity of upper- versus lower-motor neuron lesions [192]. | Ingram et al. [82],  Kale et al. [69]. |
| **Timed 25-foot Walk (T25FW)** | Administered by a trained examiner [195,196]. The participant is instructed to walk as fast and safely as possible across a marked 25-foot linear course, using an assistive device if necessary [195]. The participant is timed walking the course twice and the two trials are averaged [195]. Scoring can be expressed as time or speed [195], or as part of the Ambulatory Index [196], a 10-point scale that assesses mobility based on time and degree of assistance required during the T25FW. The T25FW is a component of the MSFC [160]. | The T25FW is related to walking performance and lower extremity function [195]. This test has strong test-retest reliability in MS across a wide range of disability levels and is sensitive to intervention effects in longitudinal studies [195]. | Nantes et al. [71]. |
| **Fatigue** | | | |
| **Fatigue Impact Scale (FIS)** | Self-report measure used to examine participants’ perceptions of how fatigue impacts their quality of life [197]. The scale is comprised of 40 items that are scored from 0 (no problem) to 4 (extreme problem), providing a total composite score of 0-160. The FIS contains subdomains that reflect perceived impact on cognitive (concentration, memory, thinking, organization of thoughts), physical (motivation, effort, stamina, coordination), and psychosocial functioning (isolation, emotions, workload, coping) (10 items/40 points each). | The FIS is a subjective measure of fatigue [197]. | White et al. [78]. |
| **Fatigue Severity Scale (FSS)** | Self-report measure that uses a series of 7-point scales to examine the severity and impact of subjective feelings of fatigue [198]. In response to each of the nine statements provided in the FSS, a rating of 1 indicates strong disagreement while 7 refers to strong agreement. A total score < 36 indicates that the individual may not be suffering fatigue, whereas > 36 suggests that one may be experiencing fatigue and should seek medical counsel [198]. | The FSS provides a subjective measure of fatigue [198]. | Bridoux et al. [66],  Perretti et al. [75]. |
| **Modified Fatigue Impairment Scale (MFIS)** | Abbreviated version of the FIS that has been adapted for persons with MS [197,199]. As for the FIS, the MFIS contains cognitive (9 items/36 points), physical (10 items/40 points), and psychosocial (2 items/8 points) subscales; however, this test only contains 21 items, and can be rated out of a total 0-84 points [197,199]. | The MFIS is a subjective measure of fatigue [197,199]. | Conte et al. [8],  Vucic et al. [87]. |

ISI, interstimulus interval.
