# Supplementary figures and images for: Transcranial Magnetic Stimulation as a Potential Biomarker in Multiple Sclerosis: A Systematic Review with Recommendations for Future Research
Source: Neural Plast. 2019 Sep 16;2019:6430596. doi: 10.1155/2019/6430596 (PMC6766108; doi:10.1155/2019/6430596)

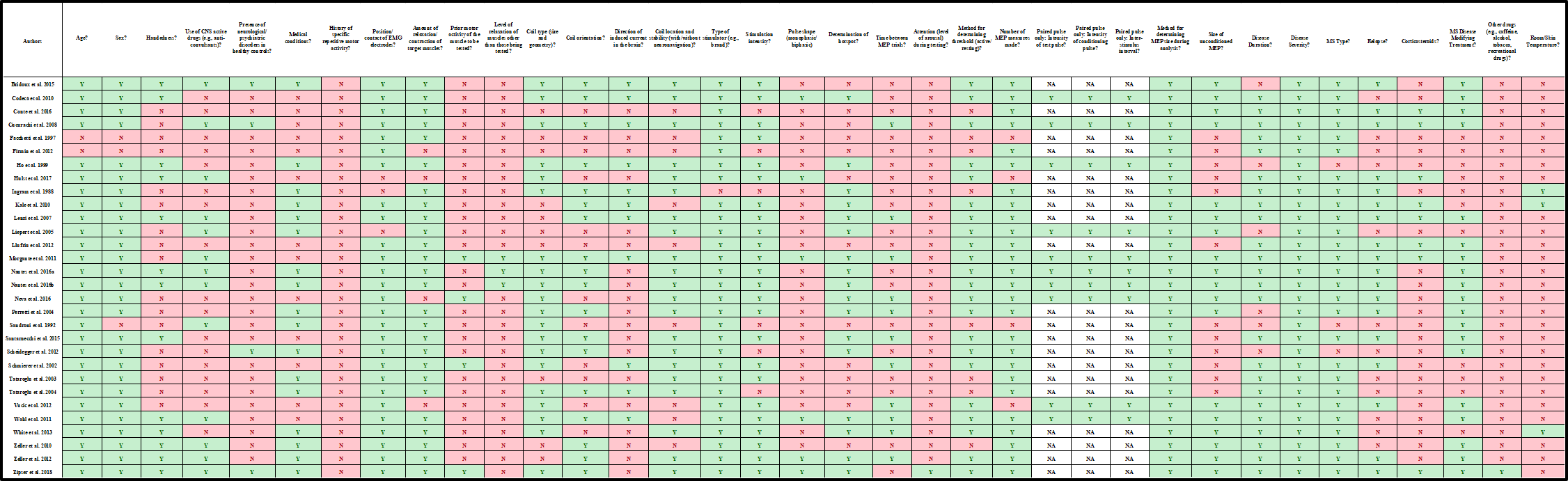

Supplement: Supplementary Materials — Figure S1: key potential confounding variable findings. In-depth descriptions of the TMS and clinical outcomes employed in reviewed studies can be found in Tables S1 and S2, respectively. See Tables S3 and S4 for results pertaining to article screening and data extraction, respectively. Original review protocol can be found in Supplemental Methods 1-2. [file 6430596.f1.zip › Supplemental Fig S1 - Confounding Variables.png]
